# Effect of the Hole Diameter Ratio (d/H) on the Web Crippling Capacity of Pultruded GFRP U-Channels Under Temperature and Loading Conditions

**DOI:** 10.3390/polym18081002

**Published:** 2026-04-21

**Authors:** Mohamed Ahmed Soumbourou, Emrah Madenci, Ceyhun Aksoylu, Yasin Onuralp Özkılıç

**Affiliations:** 1 Department of Civil Engineering, Necmettin Erbakan University, 42090 Konya, Türkiye; 2 Department of Technical Sciences, Western Caspian University, Baku 1001, Azerbaijan; 3 Department of Civil Engineering, Konya Technical University, 42250 Konya, Türkiye; 4 Department of Unique Buildings and Constructions Engineering, Don State Technical University, Gagarin Sq. 1, 344003 Rostov-on-Don, Russia

**Keywords:** pultrusion, GFRP, web crushing, high temperature, hole diameter, ITF, ETF, empirical model

## Abstract

Glass fiber reinforced polymer (GFRP) composites produced by pultrusion are increasingly used in structural applications due to their advantages such as corrosion resistance, high strength-to-weight ratio, and lightness. However, the extensive use of fibers in the longitudinal direction causes imbalance in the cross-section, leading to web crippling behavior in profiles subjected to transverse vertical forces. In this study, the influence of temperature and hole diameter on the web crippling performance of pultruded GFRP U-section profiles was investigated experimentally and analytically. The specimens were perforated with circular holes with diameters of 32–50–70 mm (diameter/Height ratio d/H = 0.23–0.36–0.50) at the web center and exposed to high temperatures of 200–250–300 °C, respectively, along with room temperature. The experiments were conducted under ITF (interior-two-flange) and ETF (end-two-flange) loading conditions. According to the results obtained, ITF configurations exhibited approximately twice the load-carrying capacity compared to ETF configurations. Due to the effect of high temperature, the web–crushing capacity showed a significant decrease of up to 44% on average in all samples when the temperature was increased from 24 °C to 300 °C. Increasing the hole diameter (and consequently the d/H ratio) led to a gradual decrease in capacity ranging from 15.7% to 56.2%; in particular, it was demonstrated that the ETF loading configuration is more sensitive to the hole than the ITF. As a result of the study, an empirical equation considering the effects of temperature and hole size was proposed, and the model’s predictions were compared with experimental results. Although the model successfully captured the general trend, the average absolute error rate in the predictions ranged between 12% and 14%, indicating improvement but not achieving ideal prediction accuracy.

## 1. Introduction

**Figure 1:**
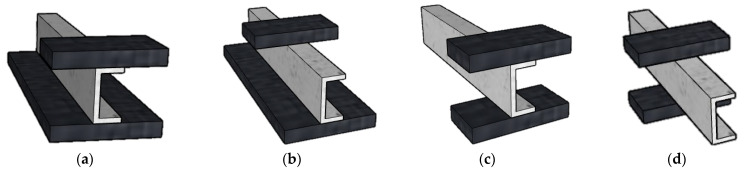
Transversal loading conditions: (**a**) EG; (**b**) IG; (**c**) ETF; (**d**) ITF.

Nowadays, the use of fiberglass composites is growing steadily and gaining momentum in the construction industry thanks to the advantages they offer to stakeholders. Created by combining different materials, each with its own advantages and disadvantages, fiberglass composites go far beyond traditional materials, which offer only simple advantages [1,2]. There are many manufacturing methods, such as hand lay-up, spray lay-up, resin transfer molding, filament winding, pultrusion, etc. [3]. Glass fibers manufactured using the pultrusion method have certain advantages that those manufactured using other methods do not have [4]. The pultrusion mechanism prevents porosity from infiltrating the material thanks to the compaction of the material during the process, which is not the case with the hand lay-up method, which is more sensitive to external weather conditions. The highly precise settings offered by the automatic pultrusion machine guarantee ideal, consistent geometry and a continuous process with unlimited fiber length without the need for human intervention. These points favor pultrusion over filament winding, hand lay-up, and other methods [5,6,7]. The large amount of (continuous) fiber used in fiberglass composites is one of the specific advantages of the pultrusion method, as other methods are limited either by fiber orientation or fiber length. Fiberglass manufactured using the pultrusion method offers various constant geometric shapes (I, U, hollow box, etc.) [8,9,10]. The distribution of the large amount of fiber in the longitudinal direction increases the mechanical strength of the material [11,12,13]. However, due to its anisotropic properties, the cross-section of pultruded GFRP does not benefit from the same characteristics as large amounts of fiber. This means that the mechanical characteristics of pultruded GFRP are weaker in the cross-section [14,15,16]. Web crippling behavior is a failure that results from the imbalance of mechanical strength characteristics in the longitudinal section and cross-section of pultruded GFRP. In web crippling tests on pultruded GFRP, the load-bearing capacity of the specimens in the transverse direction is verified by applying transverse loads in addition to small tests such as tensile and compression tests on small specimens. The types of transverse loads most commonly used by researchers are generally EG, IG, ETF, and ITF loads (Figure 1). ETF and ITF transverse loads consist of loads applied across two bearing plates at the top and bottom, either from the outside or the inside. EG and IG condition loads consist of loads applied to a bearing plate at the top with the sample placed on the ground. In the latter cases, the specimen receives a surface load on the bottom but a small surface load on the top (bearing plate surface) [17,18,19,20].

Wu et al. [21] conducted a study on pultruded GFRP channel section profiles with the aim of investigating the effects of transverse loads, specimen dimensions, and specimen length on web crippling behavior. Four different sections, each with two different lengths, were subjected to transverse loads under ETF and ITF conditions. The transverse loads caused major web buckling failures and cracking at the web–flange junctions. The effects studied were observed to have a significant impact on web crippling capacity. The latter was higher for specimens with longer lengths, lower web slenderness ratios, and when the transverse load was applied to the inside of the specimen. In another study, Wu et al. [22] focused on the effects of bearing plate length and loading conditions. Four pultruded GFRP channel sections were subjected to ETF and ITF loading conditions. Different bearing plate lengths of 20 mm, 50 mm, 100 mm, 150 mm, and 200 mm were used for the application of transverse loads. Failures at the web–flange junctions and web buckling occurred under the influence of transverse loads. An increase in bearing plate length was identified with an increase in ultimate load. Web crippling capacity was higher when loads were applied from the inside. An experimental and numerical study on the web crippling behavior of pultruded GFRP channel sections was conducted by Zhang and Chen [23]. In order to evaluate different loading conditions and bearing plate lengths, the researchers subjected specimens of pultruded GFRP channel sections to ETF, ITF, IG, and EG loading conditions, with loads applied through bearing plates of different lengths (50 mm and 150 mm). It was found that failures were more severe on samples where loads were applied through bearing plates and seated on the ground. The ultimate loads were also higher for these same specimens sitting on the ground but with the load applied from the inside. Few authors have used transverse loading conditions that we have not mentioned in the previous paragraphs, such as EOF (End-One-Flange), IOF (Interior-One-Flange), or three-point bending for evaluating the web crippling behavior of pultruded GFRP sections [24,25]. A recent study by Ye et al. [24], in which EOF and IOF loading conditions were applied to pultruded GFRP I sections, stated that the ultimate load increases with the increase in bearing plate and that the web crippling capacity is much higher under IOF loading conditions. Numerous other studies have confirmed that the ultimate load is higher when the transverse load is applied from the inside, especially when the specimen is placed on the ground, and that the ultimate load increases with the increase in bearing plate length [26,27]. A comparative study between pultruded GFRP U-channels and pultruded GFRP built-up sections, all subjected to transverse loads under ETF and ITF conditions, was conducted by Wu et al. [28]. The U-channels exhibited lower web crippling resistance compared to the built-up sections. The openings in the profiles are also one of the factors responsible for variations in web crippling capacity. In a study by Gand et al. [29] on the web crippling behavior of pultruded GFRP I sections, square and circular perforations were made on the webs of the specimens, and all specimens were subjected to ETF, ITF, IG, and EG loads. The ultimate loads were significantly higher on specimens with circular perforations. Haloi et al. [30] conducted a study on the web crippling behavior of pultruded GFRP wide-flange sections. Variable diameter openings were perforated in the centers of the sample webs and subjected to ITF transverse loading. An increase in opening diameter was observed with a decrease in ultimate load. In another study, Haloi et al. [31] drilled openings of different diameters at mid-height on the webs but at spans equivalent to the center of the bearing plate. All specimens were subjected to ETF loading conditions. Again, ultimate loads were observed to decrease with increasing openings in the specimens. High temperatures are also factors responsible for the decline in mechanical properties of pultruded GFRP materials, as reported in many studies [20,32,33].

The primary scientific problem addressed in this study is the quantitative determination of the loss of structural integrity in GFRP U-profiles manufactured using the pultrusion method when subjected to simultaneous geometric discontinuities (holes) and thermal loading. Although previous studies have primarily focused on behavior at room temperature, the interaction between localized stress concentrations around the holes and the thermal softening of the resin (matrix) has not yet been sufficiently understood [34,35]. This study tests the hypothesis that the sensitivity of the web crippling capacity to hole size increases significantly with temperature-dependent stiffness loss, and that this sensitivity differs fundamentally between end-loading (ETF) and interior-loading (ITF) boundary conditions.

## 2. Experimental Study

### 2.1. Test Specimens

**Figure 2:**
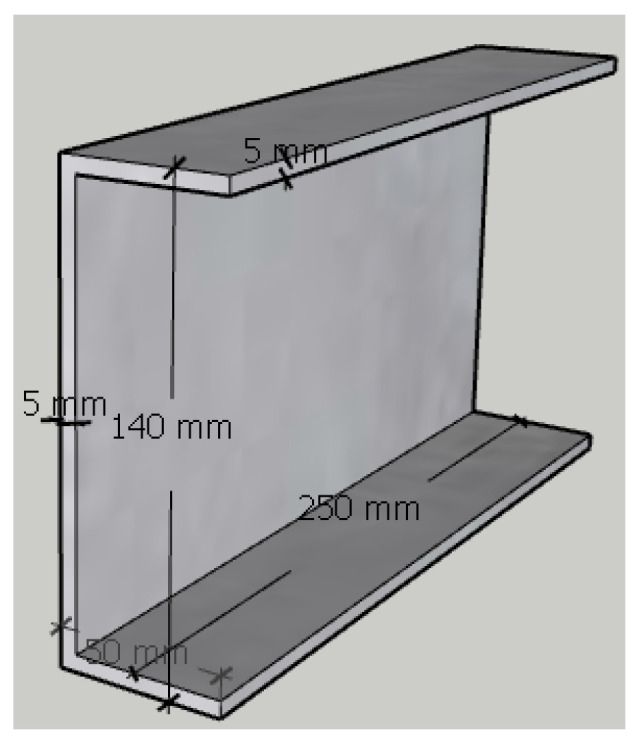
Geometric representation of the sample.

In the laboratory of Necmettin Erbakan University, a series of 32 specimens of pultruded GFRP U-channels were cut to lengths of 25 cm with widths of 5 cm, heights of 14 cm and thicknesses of 0.5 cm using a concrete saw (a saw suitable for fiberglass) (Figure 2). A total of 32 pultruded GFRP specimens were prepared for the experimental study. The specimens were divided into two main groups based on the hole locations: those with holes in the center of the web (ITF) and those with holes 50 mm from the specimen ends (ETF). Within each main group, four different subtypes were created: hole-free (reference), and those with circular voids of 32 mm, 50 mm, and 70 mm in diameter. To examine the thermal effects, the prepared samples were exposed to temperatures of 200 °C, 250 °C, and 300 °C for 1 h; samples at 24 °C were designated as the control group. The samples were tested under ambient conditions. In the test matrix presented in Table 1, the background colors (white, yellow, orange, black) represent temperature changes, while the circle sizes within the cells represent hole diameters.

All samples used in the experimental study were identified using a systematic coding method to facilitate the tracking of variables, and these data are presented in Table 1 as a visual test matrix. In this naming system, the first letter represents the profile type (U-channel), while the following abbreviations ‘ITF’ and ‘ETF’ indicate the hole positions at the center and ends of the web, respectively. The third part of the code indicates the temperature level to which the samples were exposed (24 °C, 200 °C, 250 °C, or 300 °C), while the final digits show the diameter of the circular gap in millimeters (00, 32, 50, or 70 mm). The visual design in Table 1 is structured to support this coding; the background colors of the cells (white, yellow, orange, and black) represent the temperature increase, while the sizes of the white circles within the cells represent the change in hole diameter. All samples exposed to high temperatures were kept in an oven for 1 h prior to testing to ensure thermal equilibrium. The material used in the present study was pultruded GFRP that included E-glass fibers embedded in a polyester resin matrix. By weight, the material comprised around 71% glass fiber, with the remaining 29% belonging to the polymer matrix. The reinforcement architecture has unidirectional glass fibers present in the direction of pultrusion; combined mat layers with an orientation of approximately ±45° also occupy a larger proportion, complemented by a small continuous strand mat layer in the final load. This laminated-reinforcement system is oriented such that it enhances longitudinal strength, while the matrix facilitates the transfer of loads between fibers. After thermal exposure, the specimens were allowed to cool down before mechanical testing—thus, the experiments are investigating the mechanical behavior of the pre-impregnated pultruded GFRP boards after thermal exposure, and not their sustained high-temperature loading behavior.

Due to the wide range of parameters examined (temperature, hole size, and loading conditions), a representative sample was tested for each configuration. The high degree of consistency observed in the general trends and the agreement with established studies in the literature indicate that the results reliably reflect the characteristic behavior of GFRP cross-sections.

**Table 1:** Specimen nomenclatures.

U-ITF-24-00	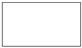	U-ETF-24-00	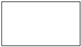
U-ITF-200-00	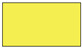	U-ETF-200-00	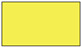
U-ITF-250-00	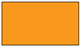	U-ETF-250-00	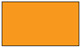
U-ITF-300-00	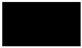	U-ETF-300-00	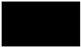
U-ITF-24-32	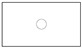	U-ETF-24-32	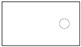
U-ITF-200-32	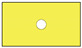	U-ETF-200-32	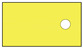
U-ITF-250-32	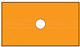	U-ETF-250-32	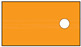
U-ITF-300-32	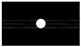	U-ETF-300-32	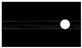
U-ITF-24-50	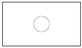	U-ETF-24-50	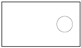
U-ITF-200-50	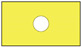	U-ETF-200-50	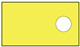
U-ITF-250-50	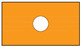	U-ETF-250-50	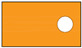
U-ITF-300-50	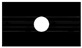	U-ETF-300-50	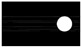
U-ITF-24-70	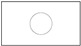	U-ETF-24-70	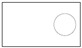
U-ITF-200-70	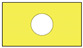	U-ETF-200-70	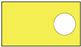
U-ITF-250-70	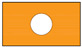	U-ETF-250-70	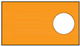
U-ITF-300-70	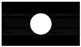	U-ETF-300-70	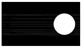

### 2.2. Loading Application

**Figure 3:**
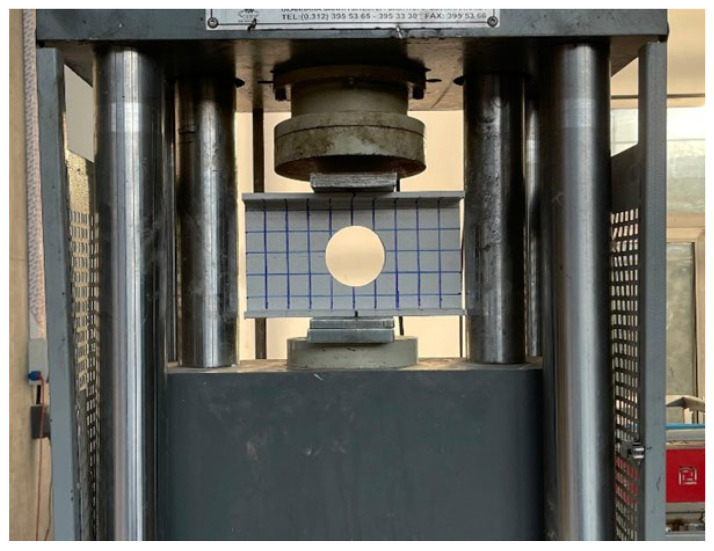
Test setup.

**Figure 4:**
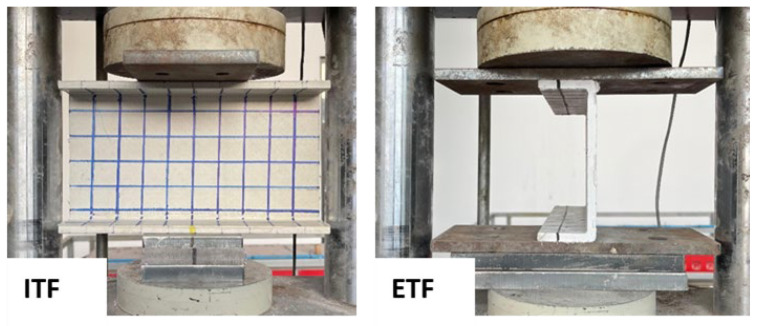
Loading condition used in the test.

The mechanical tests on the specimens were performed on a universal testing machine (Baz Makina, Ankara, Türkiye) capable of applying both tensile and compressive loads (Figure 3). The compression module of the machine was used for this study, and steel loading plates (bearing plates) were placed between the loading head and the specimens to transfer the load to them. Web crippling tests were conducted under ITF (interior-two-flange) and ETF (end-two-flange) loading conditions (Figure 4). The maximum load capacity to be applied during the tests was set at 600 kN, and the machine was programmed accordingly. To ensure that the applied transverse load was distributed homogeneously across the profile surface, loading plates of appropriate dimensions were placed on the top and bottom of the specimens. The gaps between the loading plates and the specimen surfaces were minimized to ensure full contact. The loading rate was fixed at a low value to enable detailed observation of the failure progression in the specimens. Load changes and displacement data occurring during the test were automatically recorded by the device’s data acquisition system.

### 2.3. Dynamic Mechanics Analysis (DMA)

Dynamic Mechanical Analysis (DMA) was performed to determine the viscoelastic properties of pultruded GFRP U-channel specimens. DMA specimens were cut from the web region of the profiles and prepared with dimensions of 60 × 10 × 4 mm. Tests were carried out in single cantilever mode using a Perkin Elmer DMA 8000 instrument (Shelton, CT, USA). Specimens were prepared with dimensions of 60 × 10 × 4 mm and tested at a frequency of 1 Hz, between 20 °C and 200 °C, with a heating rate of 5 °C/min. Elastic modulus (E′), loss modulus (E″), and damping coefficient (tan (δ)) values were saved, and the glass transition temperature (T_g_) and temperature-dependent behavior were evaluated.

### 2.4. Thermal Characterization—TGA-DSC

Thermogravimetric analysis (TGA) and differential scanning calorimetry (DSC) tests were performed to evaluate the thermal stability and transition temperatures of the material. The tests were realized using a Setaram–Labsys Evo instrument (Mougins, France), and approximately 10 mg samples were analyzed under a nitrogen atmosphere at temperatures ranging from 25 °C to 600 °C with a heating rate of 10 °C/min. The decomposition onset temperature was determined by TGA, and the glass transition temperature (T_g_) and possible melting transitions were determined by DSC.

### 2.5. FTIR Spectroscopy

Fourier Transform Infrared Spectroscopy (FTIR) was utilized as a method to reveal the chemical composition of the material and monitor the alterations in functional groups due to temperature modification. Measurements were taken in the 4000–500 cm^−1^ range using ATR mode with a Thermo Scientific—Nicolet iS20 instrument (Waltham, MA, USA). Analyses were obtained from both the reference sample and the heat-exposed powdered samples.

### 2.6. SEM Imaging—Scanning Electron Microscopy

Scanning Electron Microscopy (SEM) analysis was performed to investigate the fracture surfaces and microstructural properties of pultrusion GFRP U-channel samples. Using a Hitachi-SU 1510 instrument (Tokyo, Japan), fiber withdrawal, matrix cracking, and interface separations were observed on gold-plated samples. Microstructural changes, particularly those occurring after high-temperature and mechanical loading, were documented in detail.

## 3. Results

### 3.1. Effect of High Temperatures on Specimens

**Figure 5:**
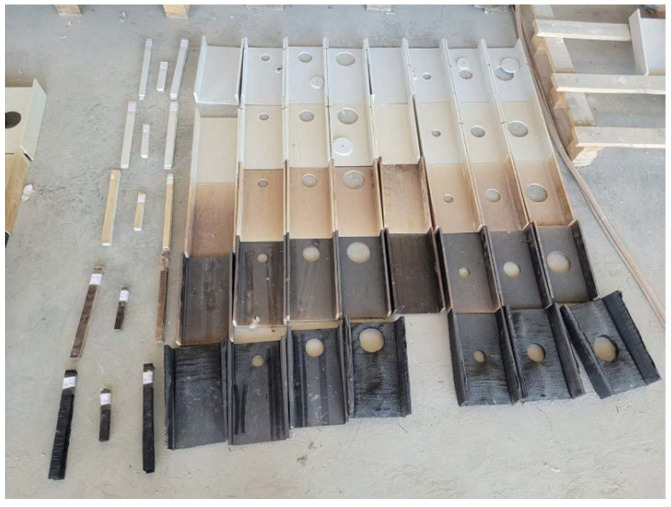
Samples subjected to different temperatures.

Pultruded GFRP U-profile samples exposed to high temperatures exhibited distinct physical responses to thermal changes. With increasing temperature, a color change was observed in the samples, progressing from white to yellow, then brown, and finally black (Figure 5). This color change is a direct indication of the thermal degradation of the polymer matrix. While no macroscopic deformation was observed in the samples at temperatures of 200 °C and 250 °C apart from the color change, it was found that at 300 °C, the samples completely darkened and microcracks began to form on their cross-sectional surfaces. As part of the experimental study, eight samples were also exposed to a temperature of 350 °C, but it was observed that these samples completely lost their structural integrity as a result of the swelling and separating of the laminate layers. Due to the excessive deformation and weakening that occurred at the web–flange joints at 350 °C, it was not possible to mechanically test these specimens. Because of this structural inadequacy, the mechanical loading tests were limited to the 300 °C limit.

### 3.2. Burn-Out Test of the Sample

**Figure 6:**
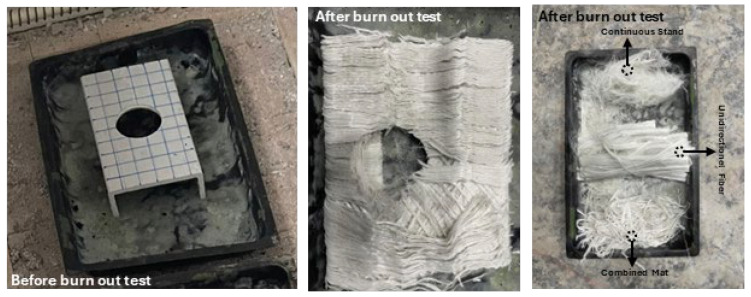
Burn-out test of the specimen.

A specimen of pultruded GFRP U-channel section with a 70 mm diameter opening in the center was prepared for the heating test in order to determine its composition. We initially weighed the specimen, which had an initial weight of 694 g. The specimen was then placed in an oven, and we set the oven to a temperature of 600 °C for 1 h. After 1 h at a temperature of 600 °C, the specimen was left with a fiber structure, the polymer components having completely evaporated. After measuring the final weight of the specimen, a total weight of 493.7 g was displayed, which means that 200.3 g of polymer contained in the specimen had evaporated. The pultruded GFRP U-channel section specimens are therefore composed of 71% fiber and 29% polymer. After the burn-out test, a fiber structure composed of three fiber phases was identified (Figure 6). The three fiber phases comprising the specimen were unidirectional fiber (outer layers), combined mat (on the secondary layers), and continuous stand mat (which was the tertiary layer) and yet another unidirectional fiber in the internal parts. The quantity of the combined mat layer was significant, representing 55% of the sample and with an orientation of approximately 45°. The continuous stand mat layer was the smallest quantity among the fibers, representing 2% of the total fiber weight. It was distributed by fine fibers with multiple orientations. The unidirectional fibers were distributed over the external (transversely on the web and longitudinally on the flange) and internal (longitudinally) parts of the specimen and represented 43% of the total fiber weight.

### 3.3. Tensile Test Results

**Figure 7:**
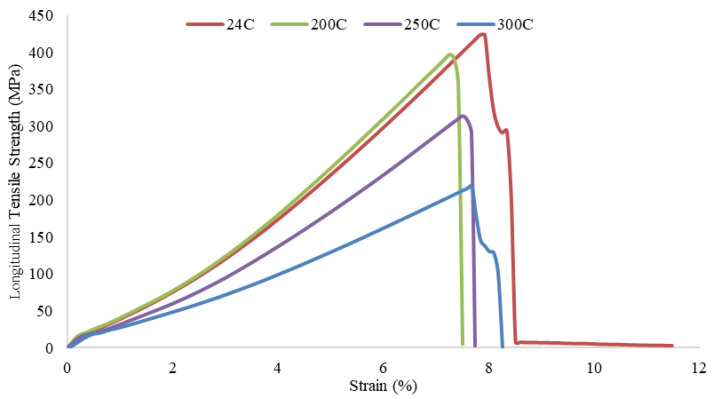
Tensile strength graph in the longitudinal direction of the specimen.

**Figure 8:**
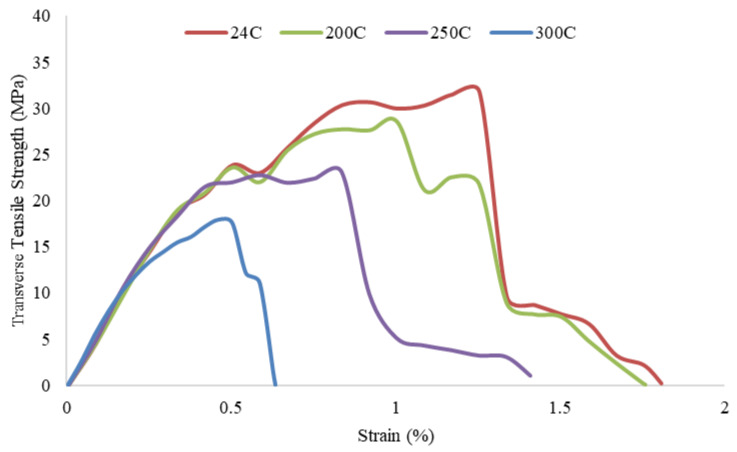
Tensile strength graph in the transversal direction of the specimen.

To determine the tensile strength of pultruded GFRP U-channel profiles, test specimens 75 mm long and 5 mm thick were prepared in accordance with ISO 527-5 [36]. The specimens were cut in the longitudinal (25 mm wide in the web direction) and transverse (15 mm wide) directions to examine the anisotropic structure of the profile. Specimens taken from both directions were exposed to temperatures of 200 °C, 250 °C, and 300 °C for one hour; specimens at 24 °C (room temperature) were used as a reference. The stress–strain curves obtained from tensile tests performed on a universal testing machine are presented in Figure 7 and Figure 8. A sharp decrease in maximum tensile strength in both directions was observed with increasing temperature. In particular, the strength loss reached its highest level in the samples exposed to 300 °C; the longitudinal strength decreased by approximately 50% compared to the reference sample. The material exhibits a strong anisotropic character depending on the fiber alignment. The tensile strength values in the longitudinal direction (approximately 215–425 MPa) exceed those in the transverse direction (approximately 18–33 MPa) by approximately 12 to 13 times across all temperature levels. This confirms that the load-bearing fibers are concentrated in the pultrusion direction. Specimens tested in the longitudinal direction (Figure 7) exhibited brittle fracture at approximately 7–8% strain values, while specimens tested in the transverse direction (Figure 8) sustained damage at much lower strain values (0.6–1.25%) due to matrix-dominant behavior. Consequently, high-temperature exposure weakened the polymer matrix, critically reducing the material’s structural performance, particularly in the transverse direction where the matrix provides load transfer.

### 3.4. Microstructure Analysis

#### 3.4.1. DMA

**Figure 9:**
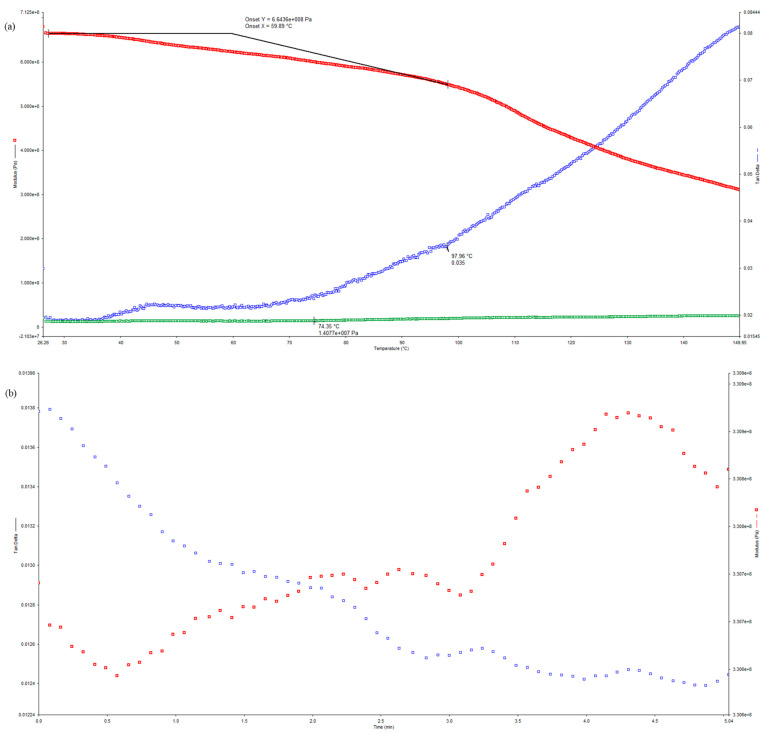

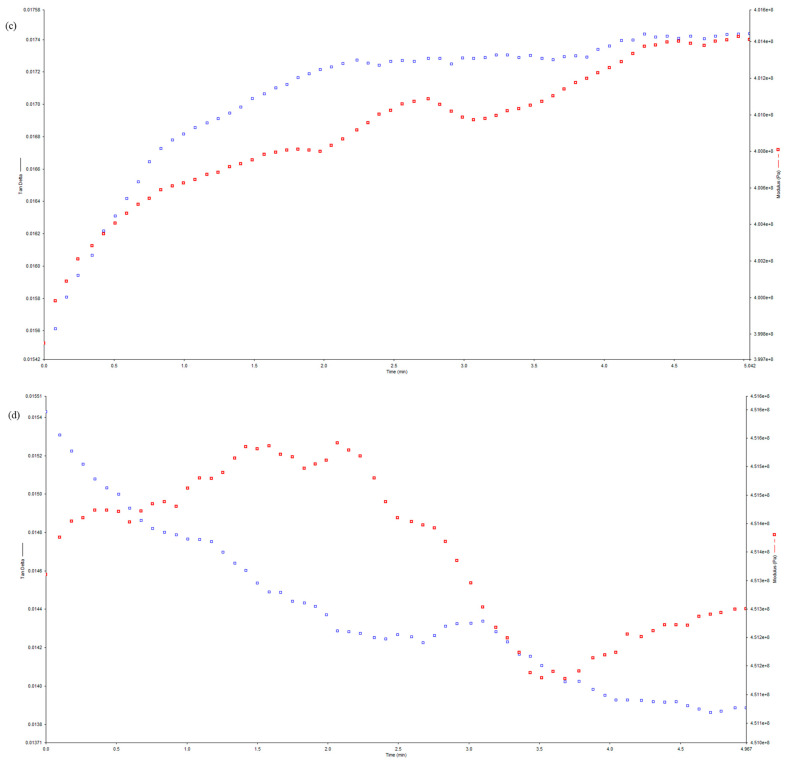

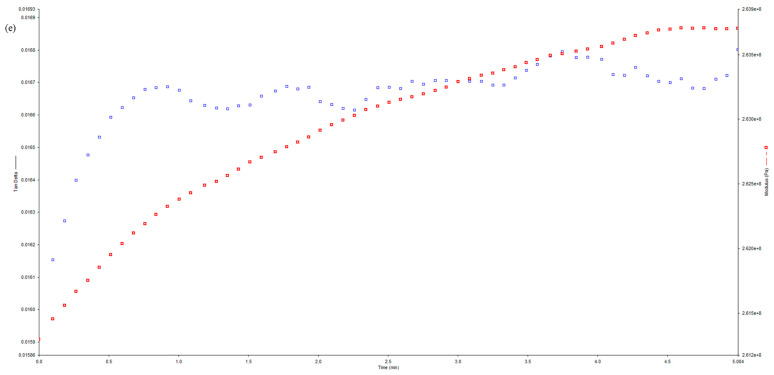
The storage modulus (E′) and damping coefficient (tan δ) curves of the samples: (**a**) U–ETF–24, (**b**) U–ETF–200, (**c**) U–ETF–250, (**d**) U–ETF–300, (**e**) U–ETF–350.

Dynamic Mechanical Analysis (DMA) was conducted in order to study the viscoelastic behavior of the pultruded GFRP U-channel, quantify the temperature dependence of stiffness degradation in the polymer matrix, and to correlate the findings with observed losses in web crippling capacity. The values of storage modulus (E′) and tan δ for the reference specimen (U-ETF-24) versus temperature from 20 to 200 °C are illustrated in Figure 9a. It was observed that a gentle decline in storage modulus was caused by an increase in temperature and hence relaxation of the polyester matrix. The onset of the decrease in stiffness began at 60 °C and beyond, brought about by chain mobility at the rubber state. From the peak of the tan δ curve, the glass transition was analyzed as 97.96 °C. This marks the region where glass matrix changes to rubber. Hereafter, the matrix’s upper limit for elastic behavior was defined. The decrease in E′ prior to Tg is moderate, whereas near and above Tg there is a sharp decrease. It indicates a significant loss of load transfer capability of the matrix phase. This is especially critical for transverse and web-dominated load cases, where it controls the matrix integrity for failure initiation.

**Figure 10:**
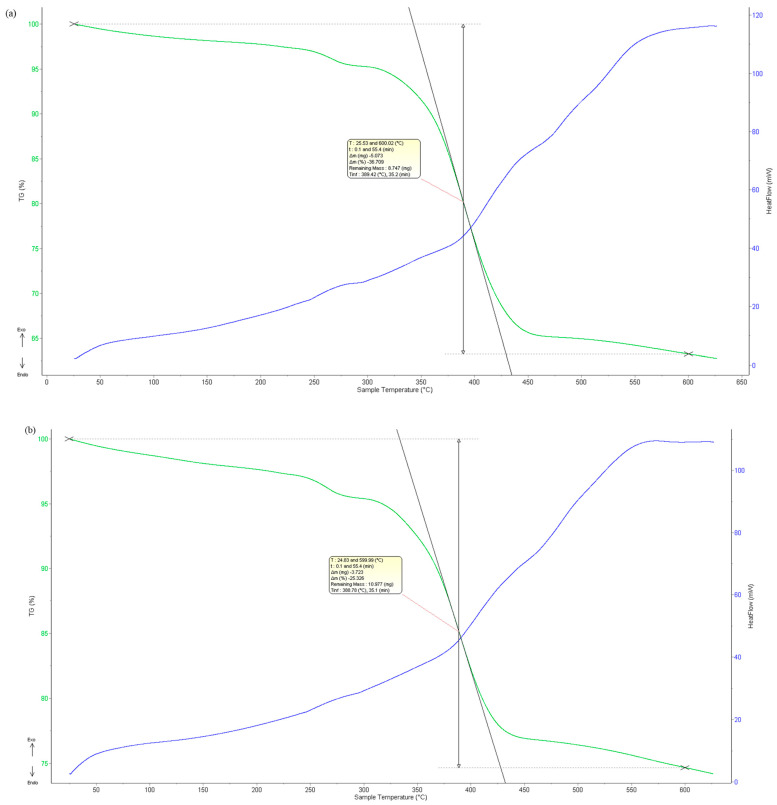
TGA–DSC curve of samples: (**a**) U–ETF-24, (**b**) U–ETF–350.

The DMA results of the thermally post-treated samples are shown in Figure 9b–e. The primary storage modulus in the specimen exposed to 200 °C, U-ETF-200 (Figure 9b), is already lower than that of the reference specimen at room temperature, signaling irreversible voids from the 1 h high-temperature exposure. Doubtless, the tan δ peak separates and becomes less sharp compared to the reference. This reflects polymer network partial degradation and a decrease in viscoelastic coherence due to aging at temperatures above Tg, rather than the entire matrix staying cool. As for the sample concerned, U-ETF-250, aging at 250 °C (Figure 9c), the storage modulus decreased still further along the temperature range. The distorted and shrunk peak of the tan δ suggests higher levels of microstructural damage within the matrix phase. Matrix softening and possible chain scission processes are believed to occur at such temperatures, further impairing the bondability of the polymer for effective stress transfer between fibers. Heating the model to 300 °C (U-ETF-300 in Figure 9d), the rapid decay in storage modulus along with a turbulent damping response was noted. The absence of a well-designed tan delta peak symptomatic of serious polymer degradation due to extensive bungle in elastic integrity means that, now, there is no way for the matrix to effectively engage in stress transfer, and fiber–matrix interfaces become the dominant factor of degradation. Noteworthy is the new way of failure observed through mechanical testing where web crushing with shear became more dominant at 300 °C. Although not actually carried out at 350 °C, for structural reasons, the DMA behavior of U-ETF-350 (Figure 9e) is indicative of a huge loss in stiffness and a very high level of damping. This situation becomes more evident with the very significant drop in E′ and high values of tan δ showing considerable degradation of the matrix and practically complete loss of any elastic functionality. These findings are confirmed by TGA–DSC (Figure 10b) where we see that the composite is on the verge of undergoing its important decomposition temperature at a temperature greater than 350 °C.

Structurally, the 44% reduction in web crippling capacity (Table 3) at a temperature of 300 °C correlates closely with the considerable reduction in storage modulus that was observed in the DMA results. As transverse stress transfer through this matrix-dominated web region is the essential mechanism of web crippling under falls rather than the normal local crushing and shear instability, degradation of E′ directly reduces the resistance of the web to local crushing and shear instability. DMA data, thus, provide a mechanistic explanation for the temperature-driven transition from web–flange junction cracking at lower temperatures (24–250 °C) to the matrix-controlled web-crushing behavior at 300 °C. In all of the DMA runs, the isothermal condition reigns reasonably uncontested as the essential parameter for pultruded GFRP U-channel high-temperature performance; it crystallizes a point that the thermal degradation of the matrix, not the weakening of fiber strength, has that key remarking role. Determining the storage modulus loss at the glass transition more so brings various related capacities down.

#### 3.4.2. TGA-DSC

TGA and DSC analyses were performed to investigate the thermal stability and decomposition behavior of the materials. The TGA–DSC curve obtained for the reference sample, U-ETF-24, is presented in Figure 10a. A total mass loss of 36.71% was observed whilst heating the sample from 25.5 °C to 600.0 °C. The majority of this loss occurred in the main decomposition region, which starts at approximately 389.42 °C and progresses rapidly. This temperature represents the critical threshold at which the sample undergoes thermal decomposition. The clear endothermic response being the strongest in the same region on the DSC curve reveals that the matrix phase has turned to ash and there has been a considerable increase in heat flux.

TGA–DSC results for the U-ETF-350 sample, heated to 350 °C, are given in Figure 10b. A similar degradation trend was observed in this sample, but the total mass loss remained at 25.23%. The onset temperature of degradation was again approximately 388.78 °C, almost identical to the reference sample. However, the lower total mass loss suggests the presence of residues with higher thermal stability in the structure, or that some components have already partially decomposed during the heat exposure. The endothermic response in the DSC curve is less sharp, and the increase in heat flux is slower. This suggests that the matrix may have been structurally weakened due to previous heat exposure.

In both samples, the rapid thermal degradation region occurring around 389 °C represents a limiting factor in terms of the maximum service temperature at which the composite can be used. The observation of significantly less mass loss and less energy absorption in the sample exposed up to 350 °C indicates that the composite undergoes some permanent microstructural changes at these temperature levels and that the DSC signal is weakened.

A high mass loss evident in TGA with respect to the polymer content found by the burn-off test could be used to account for moisture condensation as well as other volatile components contained within the polymer matrix.

#### 3.4.3. FTIR

**Figure 11:**
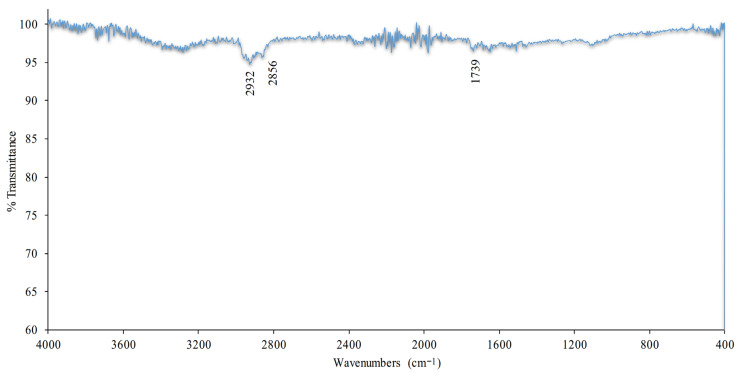
FTIR spectrum of sample U-ETF-24.

FTIR analysis was performed to determine the chemical bond structures and functional groups of the U-ETF-24 sample produced by the pultrusion method. In the FTIR spectrum presented in Figure 11, the characteristic absorption bands of the organic matrix components of the sample are clearly visible. The strong absorption band around 1730 cm^−1^ corresponds to ester carbonyl groups (C=O), confirming the presence of a polyester matrix. Bands observed in the range of 1250–1150 cm^−1^ indicate C–O stretching vibrations. Peaks between 1450 and 1375 cm^−1^ originate from bending vibrations of methyl (–CH_3_) and methylene (–CH_2_–) groups. Furthermore, the broad band around 3400 cm^−1^ may belong to O–H groups, suggesting the presence of moisture or hydroxyl groups in the sample. This FTIR spectrum demonstrates that the basic components of the U-ETF-24 sample are compatible with a polymeric matrix and retain the characteristic structures of the resin used.

#### 3.4.4. SEM-EDS

**Figure 12:**
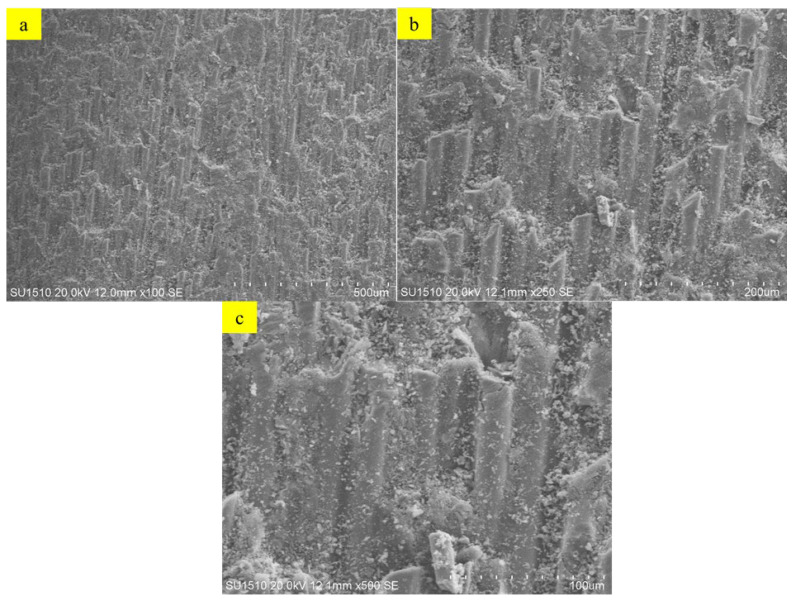
SEM images of sample U-ETF-24 (low temperature, 24 °C); (**a**) ×100 SE; (**b**) ×250 SE; (**c**) ×500 SE.

**Figure 13:**
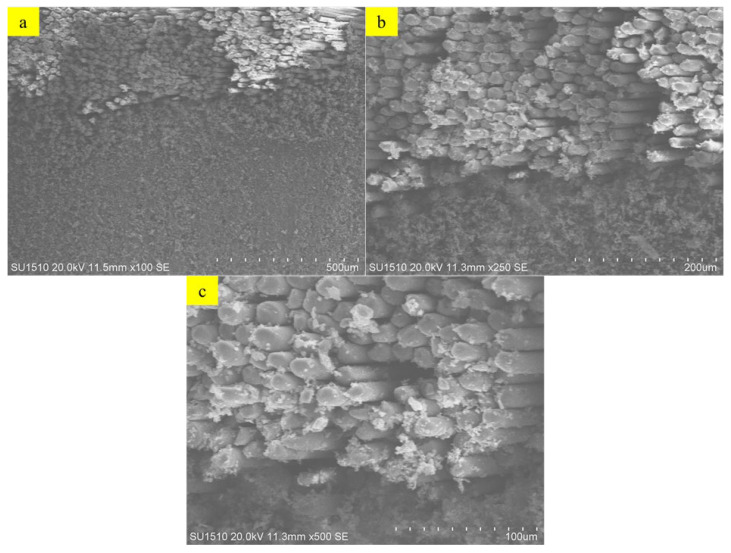
SEM images of sample U-ETF-200; (**a**) ×100 SE; (**b**) ×250 SE; (**c**) ×500 SE.

Scanning Electron Microscopy (SEM) analysis was performed to evaluate the microstructural properties of U-ETF composite samples. This analysis provides a comparative analysis of the surface morphologies of samples processed at different temperatures and allows for the evaluation of the effects of the manufacturing process on microstructural integrity. Figure 12a–c present SEM images of the U-ETF-24 sample produced at 24 °C. These samples show a highly uniform and coherent fiber–matrix interface. The resin homogeneously enveloped the fibers, and no porous structure was observed. The matrix appears to adhere well to the fiber surface, indicating that the pultrusion process occurred at a low temperature with appropriate parameters and that the composite structure possesses a robust bonding interface. No visible cracks, pores, or separations are present in the microstructure. This suggests a high level of mechanical integrity of the sample. On the other hand, Figure 13a–c show SEM images of the U-ETF-200 sample processed at 200 °C. In these samples, some separations, micro-voids, and partial breaks in the fiber–matrix bond structure were observed in the interface regions. Some areas where the resin receded and complete encapsulation could not be achieved were detected on the fiber surfaces. This indicates that high temperature negatively affects interface integrity by influencing the viscosity and flow characteristics of the resin. At the same time, traces of microcracks due to thermal stresses were also found in certain regions of the microstructure. These observations reveal that the production temperature has a direct effect on the microstructure and should be considered as a critical optimization parameter.

**Figure 14:**
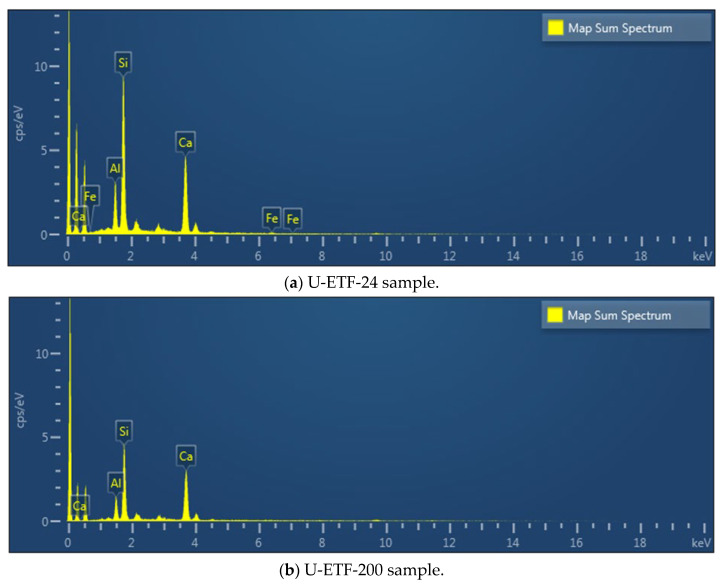
EDS spectrum and elemental distribution of samples.

Energy Dispersive X-ray Spectroscopy (EDS) analysis was performed in conjunction with SEM to determine the surface chemical composition of U-ETF samples produced by the pultrusion method. Analyses were carried out on both a reference sample obtained at 24 °C and a sample exposed to 200 °C. The obtained spectra and elemental percentages are presented in Figure 14a and Figure 14b, respectively. EDS analysis of the U-ETF-24 sample revealed that the most abundant elements were carbon (49.50%) and oxygen (34.53%), confirming that the polymeric matrix structure of the sample consists predominantly of organic components. In addition, silicon (7.00%), calcium (6.34%), and lower amounts of aluminum (2.41%) and iron (0.23%) were also detected. The presence of silicon and calcium suggests the presence of glass fiber reinforcement and possible fillers. Iron was present in trace amounts, likely due to production or environmental contamination. A similar chemical composition profile was observed in the EDS spectrum of the U-ETF-200 sample, with carbon (41.83%) and oxygen (38.12%) again being the dominant elements. However, compared to the 24 °C sample, the carbon ratio decreased while the oxygen ratio increased. This can be explained by oxidation and potential matrix decomposition occurring in the polymer matrix structure. In addition, increases were observed in silicon (7.81%) and calcium (9.50%) ratios, and the aluminum (2.74%) ratio increased only slightly. These increases indicate that the mineral phases become more prominent on the surface as a result of the decomposition of the organic matrix with increasing temperature. In both analyses, the Si and Ca elements associated with glass fiber reinforcement are present in remarkable proportions, confirming the inorganic characteristics of the fiber used. The change observed in the surface chemical composition of the matrix in the sample exposed to 200 °C is considered a critical finding that could directly affect microstructure and mechanical performance.

These findings quantitatively demonstrate the effect of different temperatures on surface chemistry and highlight the importance of chemical stability, which plays a decisive role in composite performance (Table 2).

### 3.5. Failures and Load–Displacement of Specimens

#### 3.5.1. Specimens Subjected to Room Temperature and ETF Loading Conditions

**Figure 15:**
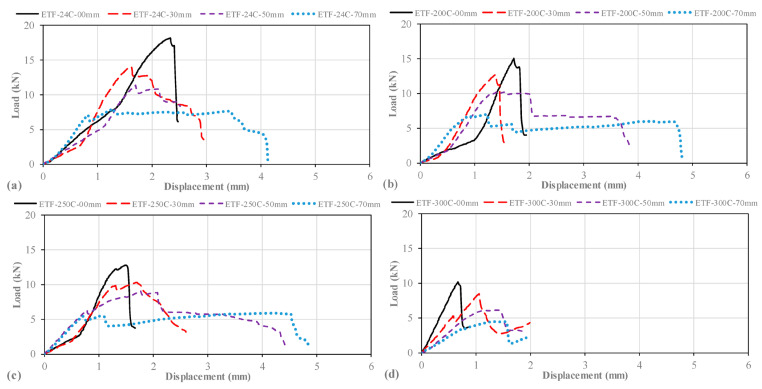
Load–displacement curve of samples loaded under ETF and exposed to: (**a**) 24 °C, (**b**) 200 °C, (**c**) 250 °C, (**d**) 300 °C.

**Figure 16:**
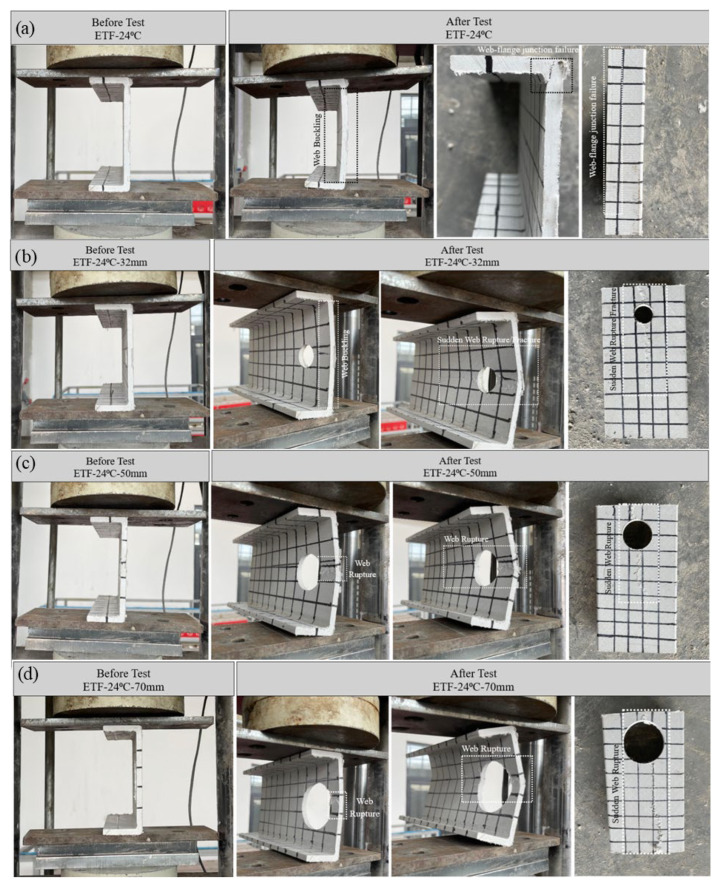
Condition of samples before and after loading: (**a**) ETF-24-00, (**b**) ETF-24-32, (**c**) ETF-24-50, (**d**) ETF-24-70.

Pultruded GFRP U-channel sections at room temperature (24 °C) were examined as baseline performance data for comparing thermal effects under end-two-flange (ETF) loading. The main factor differentiating the capacity in this group was the change in the diameter (D) of the web holes. Experimental results confirmed that specimens not exposed to thermal effects achieved the highest ultimate capacities according to the relevant hole sizes (Figure 15a). The dominant failure modes identified were web–flange junction cracking and web crippling (Figure 16a–d).

The unperforated reference profile (D = 0) reached the highest ultimate load of 18.17 kN (Figure 15a). In the initial stage, significant web buckling was observed up to approximately 9 kN. Beyond this load level, secondary cracks began at an angle of approximately 45° at the upper web–flange junction just before ultimate structural failure (Figure 16a). The load–displacement curve collapsed at the ultimate load of 18.17 kN, exhibiting a sharp peak indicating relatively brittle structural failure (Figure 15a). A further analysis of the load–displacement curve (Figure 15a) for the intact specimen reveals an important observation. It can be seen that the stiffness increases after the initial load plateau of approximately 9 kN, reaching the maximum load-bearing capacity. This can be explained by the initial web buckling observed due to the bearing plate contact locally disappearing and the damage along the web–flange junction (beam) length.

The observed capacity reductions were directly proportional to the ratio of the hole diameter (D) to the section height (H) (D/H = 0.23, 0.36, 0.50). The specimen with a 32 mm diameter hole (D/H = 0.23) reached a final load (ultimate load) of 13.97 kN. The load–displacement curve of this specimen showed a linear progression with excessive web buckling followed by slight cracking at the mid-height of the web (Figure 15a and Figure 16b). A critical observation in this specimen was that a significant increase in stiffness was detected after a load of approximately 3 kN, followed by a sudden drop in load due to sudden web fracture at the maximum load. This temporary increase in stiffness can be interpreted as the flanges beginning to engage and actively resist the failure mechanism. Furthermore, this situation can be seen as an effort to balance the onset of damage in the web. However, the pre-existing perforation in the web led to the final failure mode being web rupture/fracture starting from the region close to the end-support area. This is the exact opposite of the web–flange junction failure observed in the unperforated reference specimen. This clearly demonstrates that the presence of the web opening critically altered the controlling failure mode from junction failure to localized web fracture.

The ultimate load in the 50 mm diameter specimen (D/H = 0.36) decreased to 11.38 kN (approximately 11 kN). Significant web rupture was observed at the mid-height of the web near the 25 mm tip. This damage spread toward the center of the web, but the crack intensity noticeably weakened as it moved away from the high-stress zone around the hole (Figure 15a and Figure 16c).

The 70 mm diameter perforated specimen (D/H = 0.50) recorded the lowest capacity of 7.97 kN in the 24 °C group, indicating a decrease of approximately 56% compared to the unperforated specimen. This specimen exhibited approximately linear elastic development up to a vertical load of 7 kN. Damage progression began with the first crack at the mid-height of the web and continued until the web completely fractured at the ultimate load of approximately 8 kN (Figure 16d). Notably, this specimen recorded the largest total displacement of 4 mm sustained from the elastic phase to the plastic phase (Figure 15a). However, this significant total displacement does not represent the ductile behavior of the material; rather, it stems from the complete structural collapse and fracture of the web region under load. The damage observed in the 70 mm diameter specimen is explained by brittle and sudden fractures following severe web buckling. Therefore, the governing failure mechanism is localized web rupture, triggered by stress concentration, indicating rapid and non-ductile failure. In other words, the initial crack started in the small section (ligament) between the hole edge and the 15 mm tip but spread across the entire tip width at the final loading moment. These findings collectively confirm that increasing the hole diameter not only reduces the effective web area but also critically increases the stress concentration around the tip support area, accelerating the onset of the web damage mode.

#### 3.5.2. Specimens Subject to 200 °C and ETF Loading Conditions

**Figure 17:**
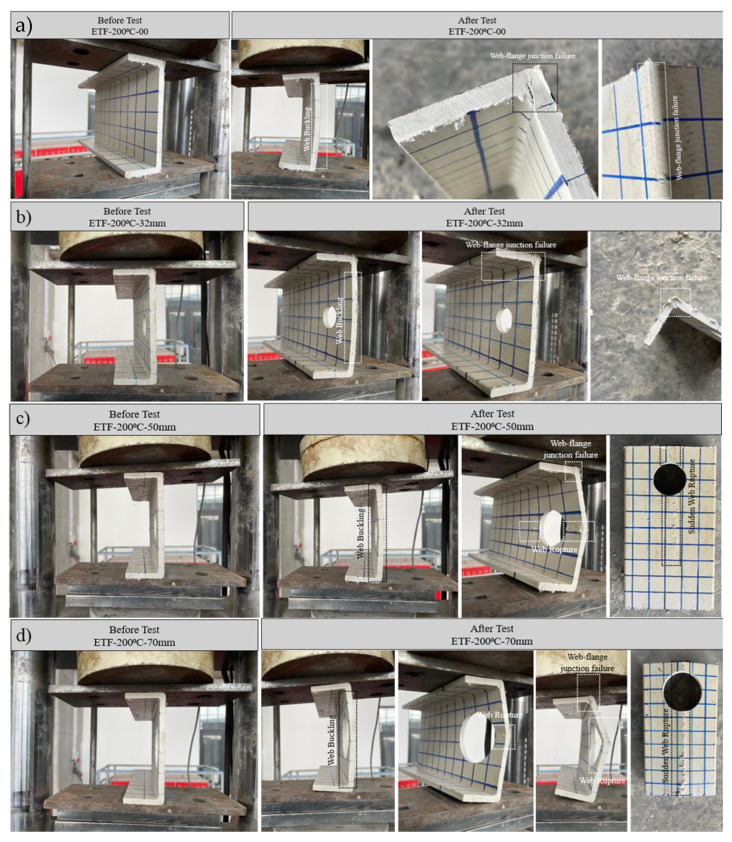
Condition of samples before and after loading: (**a**) ETF-200-00, (**b**) ETF-200-32, (**c**) ETF-200-50, (**d**) ETF-200-70.

Under end-two-flange (ETF) loading conditions, specimens exposed to 200 °C temperatures failed primarily due to a combination of web–flange junction cracking and web ruptures. This thermal exposure caused a net decrease in both stiffness and ultimate capacity compared to specimens at room temperature (24 °C) (Figure 17a). All specimens in this group exhibited elastic linear displacement up to approximately 1 mm vertical displacement (Figure 15b).

For the unperforated specimen (D = 0 mm), initial web buckling was observed at the start of loading. The profile reached an ultimate load of 15.06 kN; at this point, an initial fracture occurred locally at the web–flange junction (Figure 17a). The load–displacement curve exhibited a parabolic shape, and the subsequent sharp peak indicated a non-progressive, brittle failure mode with limited crack propagation along the length.

A similar but less pronounced capacity reduction was recorded in the specimen with a 32 mm diameter hole. This specimen exhibited an approximately linear and elastic response up to an ultimate load of approximately 12.69 kN (approximately 13 kN), followed by fracture concentrated at the web–flange junction with partial web buckling (Figure 17b).

The 50 mm diameter perforated specimen initially exhibited web buckling up to an ultimate load of approximately 10.25 kN. Beyond this load, damage progressed in two stages: an initial fracture at the web–flange junction and, immediately following, a secondary fracture localized beyond the tip of the hole at the mid-height of the web. It was observed that the resulting cracks spread along the web length across the region covered by the bearing plate width, primarily due to the horizontal components generated under vertical transverse loading (Figure 17c).

Finally, the specimen with the largest opening (70 mm diameter) exhibited the lowest capacity (7.00 kN) in this group. This specimen also exhibited initial web buckling up to the ultimate load, followed by initial cracking at the web–flange junction and then a structural fracture at the mid-height of the web (Figure 17d). This specimen exhibited the greatest total displacement compared to the other 200 °C specimens and was observed to have deep cracks extending along the entire length of the specimen at the mid-height of the web. However, it should be noted that this significant total displacement does not represent the ductile behavior of the material.

#### 3.5.3. Specimens Subjected to 250 °C and ETF Loading Conditions

Samples exposed to 250 °C under end-two-flange (ETF) loading conditions exhibited damage patterns qualitatively similar to those at 200 °C, but significant differences were observed in their ultimate load capacities and post-peak behavior due to accelerated thermal degradation of the matrix (Figure 15c).

**Figure 18:**
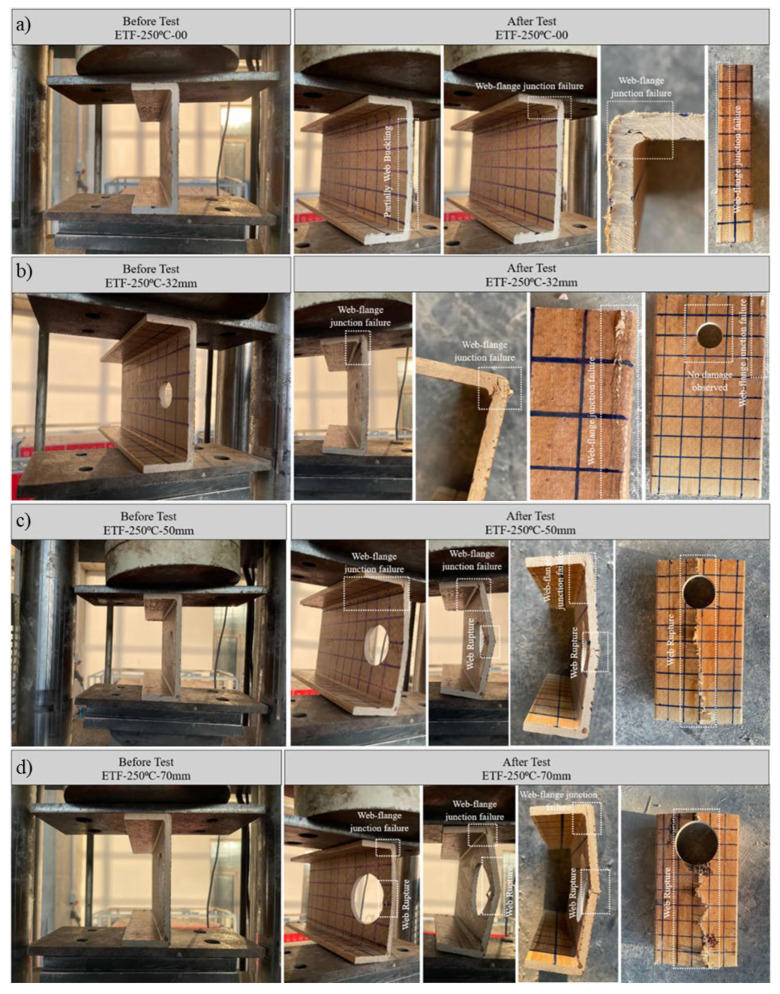
Condition of samples exposed to 250 °C before and after loading: (**a**) ETF-250-00, (**b**) ETF-250-32, (**c**) ETF-250-50, (**d**) ETF-250-70.

The unperforated reference specimen (D = 0 mm) initially exhibited partial web buckling up to an ultimate load of approximately 12.79 kN (approximately 13 kN) (Figure 18a). Beyond this peak load, the specimen is fractured at the web–flange junction (Figure 18a). Notably, this specimen achieved the least ultimate vertical displacement within the 250 °C series. Cracking initiated at the joint under the directly applied load and propagated along the beam but did not spread significantly along the web length; this indicates that the damage was controlled by stress concentration under the applied vertical load.

For the specimen with a 32 mm diameter (D/H = 0.23) opening, linear and elastic behavior was maintained up to a load of 9 kN. From this point onwards, the onset of fracture was observed at the web–flange junction. This damage intensified with increasing load until structural failure occurred at the joint under an ultimate load of 10.02 kN (approximately 10 kN) (Figure 18b).

More severe damage progression was observed in specimens with larger openings. Both the 50mm (D/H = 0.36) and 70 mm (D/H = 0.50) diameter specimens initially showed signs of damage at the web–flange junctions. However, the dominant damage mechanism shifted towards the web region. At ultimate loads of 9.06 kN for the D/H = 0.36 specimen and 5.87 kN for the D/H = 0.50 specimen, the web sections fractured cleanly at mid-height. These mid-height cracks were large-scale and extended along the entire length of the specimen (Figure 18c,d). The significant capacity reduction in the D/H = 0.50 specimen highlights the critical interaction between the geometric weakening caused by the large hole and the softening of the material at 250 °C.

#### 3.5.4. Specimens Subjected to 300 °C and ETF Loading Conditions

Specimens exposed to 300 °C exhibited a distinct transition in damage mechanisms compared to the low-temperature groups. While damage at the web–flange junctions persisted, web crippling became the dominant damage mode due to severe thermal degradation of the polymer matrix. Additionally, minor blistering or swelling of the surface coating was observed due to high thermal exposure.

**Figure 19:**
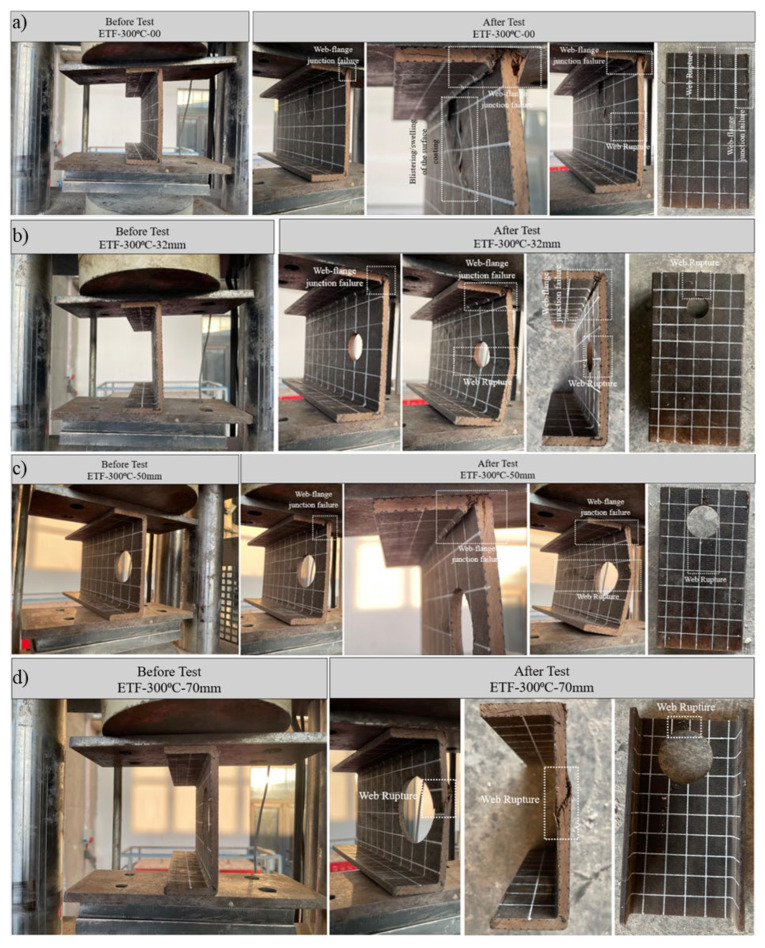
Condition of samples exposed to 300 °C before and after loading: (**a**) ETF-300-00, (**b**) ETF-300-32, (**c**) ETF-300-50, (**d**) ETF-300-70.

The undeformed reference specimen (D = 0 mm) initially exhibited approximately linear elastic behavior up to an ultimate load of 10.19 kN (approximately 10 kN) (Figure 15d). Unlike the low-temperature tests where junction damage was predominant, this specimen failed due to a combination of web–flange junction fracture and, more importantly, web crippling (Figure 19a). Significant displacements were recorded at the upper web–flange junction, along with cracks extending over a length equivalent to the width of the load-bearing plate (100 mm). This web damage spread from the center of the web and inward from the edge, indicating a loss of global cross-sectional stability.

For the 32 mm diameter (D/H = 0.23) perforated specimen, the load increased linearly up to approximately 5 kN, then decreased slightly before increasing linearly again. Finally, when it reached 8.46 kN, the test ended with sudden failure. The damage started at the web–flange junction at a 45° angle and spread across the width of the bearing plate. This was followed by web buckling/rupture damage at the mid-height at the ultimate load (Figure 19b).

The 50 mm diameter specimen (D/H = 0.36) exhibited a distinct pseudo-ductile response with greater vertical displacement (approximately 1.4 mm) at a reduced ultimate load of 6.13 kN (approximately 6 kN). The specimen initially fractured deep within the web–flange junction, followed by a tear associated with buckling at the mid-height of the web. Crack propagation lengths remained consistent with previous specimens, extending across the width of the bearing plate (Figure 19c).

The specimen with the largest hole diameter (70 mm, D/H = 0.50) exhibited a unique damage mode. This specimen did not primarily fracture at the web–flange junction; instead, at an ultimate load of approximately 5 kN, a shear failure occurred in the remaining narrow ligament/region (ligament, ~15 mm) between the hole and the beam end (Figure 19d). This damage resulted from severe web buckling and caused the crack to propagate to a length slightly exceeding the width of the load-bearing plate. Furthermore, it was observed that specimens exposed to 350 °C had the lowest load-carrying capacity.

When a general comparison of tests conducted under the ETF loading configuration is made, it reveals that the interaction between temperature increase and web openings fundamentally alters the load-bearing mechanism of the specimens. At room temperature (24 °C) and temperatures between 200 and 250 °C, damage generally develops as localized cracks in areas where stress concentration is intense (web–flange junction). At the critical temperature threshold of 300 °C, with the loss of matrix cohesion, global web crippling and shear failure become dominant. Particularly in specimens with a D/H = 0.50 (70 mm) ratio, the combination of geometric weakness and thermal degradation led to dramatic drops in capacity and sudden pseudo-ductile failures. These results demonstrate that under ETF loading, temperature not only reduces strength but also shifts the damage mode character from brittle fracture to matrix-controlled crushing/shearing.

#### 3.5.5. Specimens Subjected to Room Temperature and ITF Loading Conditions

**Figure 20:**
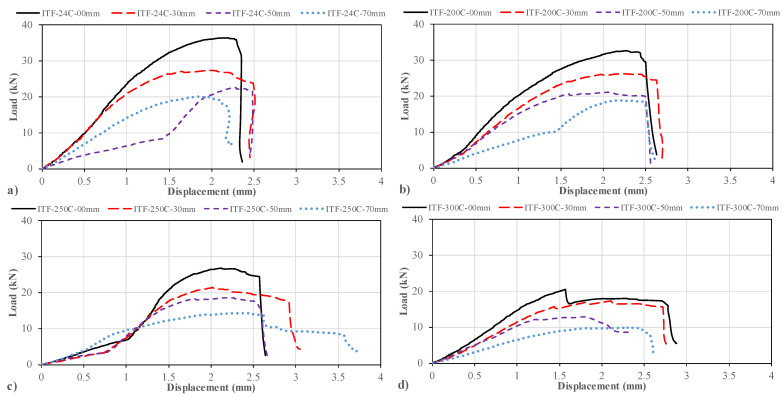
Load–displacement curve of samples loaded under ITF and exposed to: (**a**) 24 °C, (**b**) 200 °C, (**c**) 250 °C, (**d**) 300 °C.

At room temperature of 24 °C under the interior-two-flange (ITF) loading configuration, the primary damage mechanism identified for pultruded GFRP U-channels was web crippling. As seen in the load–displacement curves (Figure 20a), all specimens exhibited consistent behavior and reached their ultimate loads at vertical displacements between 2 mm and 2.5 mm.

**Figure 21:**
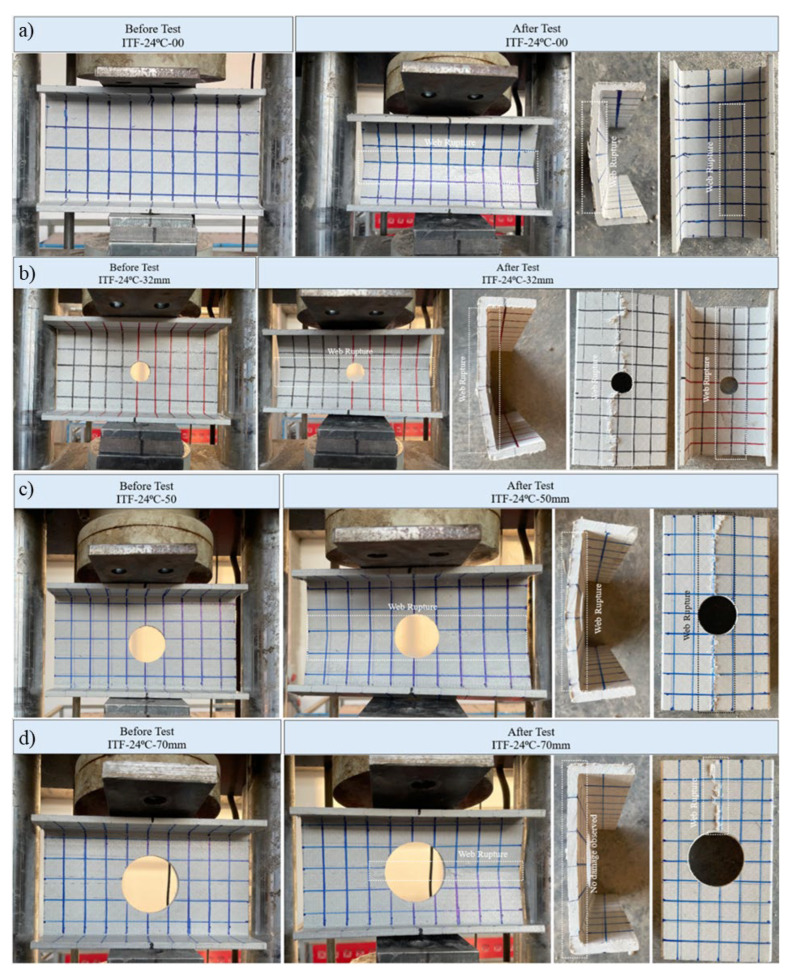
Condition of samples exposed to room temperature before and after loading: (**a**) ITF-24-00, (**b**) ITF-24-32, (**c**) ITF-24-50, (**d**) ITF-24-70.

When examining the non-perforated reference specimen (D = 0 mm), it exhibited a linear elastic response up to approximately 25 kN and failed at a peak load of 36.44 kN (Figure 20a). Damage occurred in the form of a longitudinal fracture at the web mid-height (Figure 21a). Visual inspection revealed a slight crack starting at the center of the inner web surface and extending longitudinally to approximately 10 cm. The outer surface also fractured at the web mid-height; however, since there was no thermal discoloration at room temperature, the visibility of the crack was lower compared to the high-temperature specimens.

The addition of web perforations has caused a gradual decrease in load-carrying capacity. The specimen with a 32 mm diameter hole (D/H = 0.23) maintained its linear elastic behavior up to approximately 25 kN. The first signs of damage appeared as small cracks at the web–flange junction. At a final load of 27.38 kN, the specimen experienced a significant web crippling failure at mid-height. This fracture propagated along the entire length of the profile and was clearly visible on the outer web surface (Figure 21b).

For the 50 mm diameter (D/H = 0.36) specimen, linear behavior was observed up to approximately 9 kN, beyond which initial fractures began to develop at the web mid-height. The specimen failed at a final load of 22.66 kN. The damage mode was a structural failure at the web mid-height, with the crack propagating along the entire length of the specimen due to the reduced effective web area (Figure 21c).

The specimen with the largest hole diameter (D/H = 0.50, D = 70 mm) failed at a final load of 20.11 kN with a vertical displacement of approximately 1.9 mm. Although it represented the lowest capacity in the group due to geometric reduction, the specimen still exhibited significant residual resistance. Unlike the small-hole specimens, where the crack propagated along the entire length, the damage in the 70 mm specimen occurred in the form of a mid-height fracture, where crack propagation was asymmetric; i.e., it propagated primarily from one side of the hole toward the beam end (Figure 21d).

#### 3.5.6. Specimens Subjected to 200 °C and ITF Loading Conditions

Specimens subjected to interior-two-flange (ITF) loading conditions at 200 °C failed primarily due to web crippling. Although the observed damage progression was qualitatively similar to that at room temperature, thermal softening led to changes in the load–displacement characteristics (Figure 20b).

**Figure 22:**
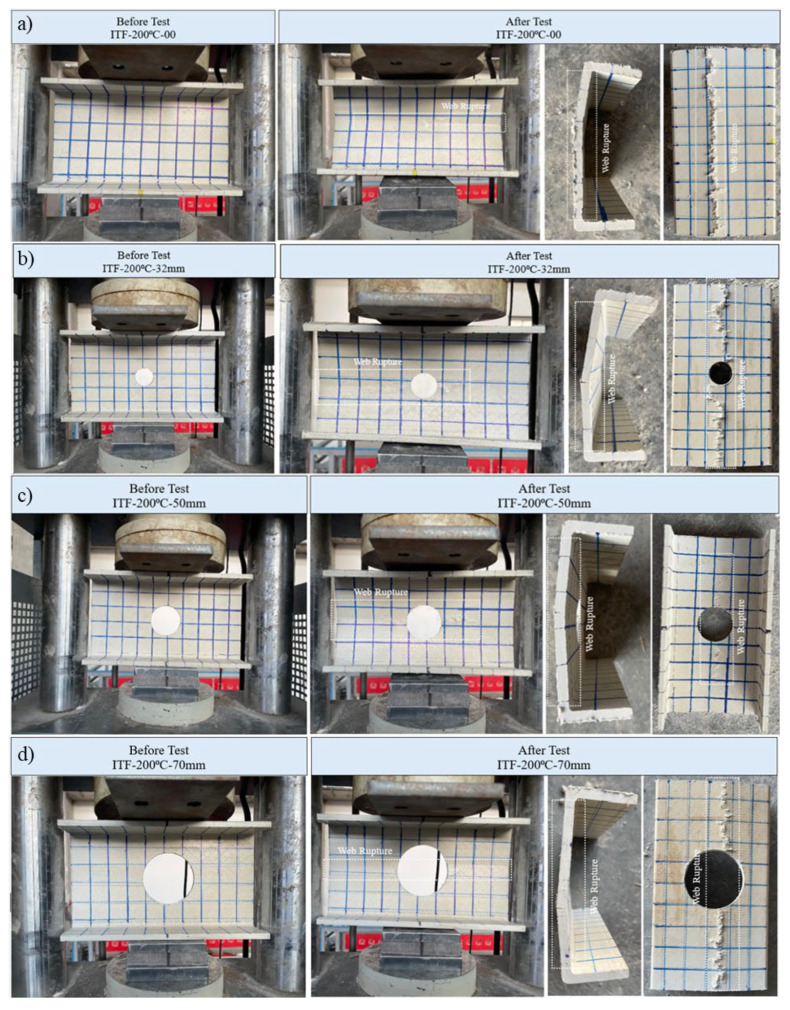
Condition of samples exposed to 200 °C before and after loading: (**a**) ITF-200-00, (**b**) ITF-200-32, (**c**) ITF-200-50, (**d**) ITF-200-70.

The reference sample without holes (D = 0 mm) exhibited web buckling up to a final load of 32.67 kN (approximately 33.00 kN). An initial crack formed at the mid-height of the web during the loading phase. At the ultimate load, the specimen fractured cleanly from this initial point, and the final crack propagated along the entire length at the mid-height of the web (Figure 22a).

The specimen with a 32 mm diameter hole (D/H = 0.23) exhibited approximately linear elastic behavior up to an ultimate load of 26.25 kN. Notably, this specimen exhibited greater ultimate vertical displacement compared to other configurations in this group. A slight crack was observed at the web mid-height at the start of loading; however, at the ultimate load, a complete fracture occurred at this position and extended along the entire length of the profile (Figure 22b).

For a sample with a 50 mm hole diameter (D/H = 0.36), linear elastic behavior is maintained up to approximately 20 kN of vertical load. Beyond this point, the load–displacement curve exhibited a distinct displacement plateau, where the load remained relatively constant while the displacement progressed from 1.5 mm to 2.5 mm. This pseudo-ductile stage continued until a final load of 21.12 kN was reached. During this constant load stage, small cracks initiated at the mid-web and propagated horizontally until final failure (Figure 22c).

The specimen with the largest hole diameter (70 mm, D/H = 0.50) exhibited linear elastic behavior up to an approximately 10 kN load. From this point onward, the specimen showed significant permanent resistance with lower relative displacement and reached a final load of 18.91 kN (approximately 19.00 kN). At the mid-height of the specimen, a definite web crippling failure was observed, with a fracture propagating along the entire length of the web (Figure 22d).

#### 3.5.7. Specimens Subjected to 250 °C and ITF Loading Conditions

Under ITF loading conditions at 250 °C, damage modes became more complex, involving both large-scale crippling at the web mid-height and damage at the web–flange junction. The load–displacement behavior (Figure 20c) revealed different stiffness phases due to thermal softening followed by compaction/hardening.

**Figure 23:**
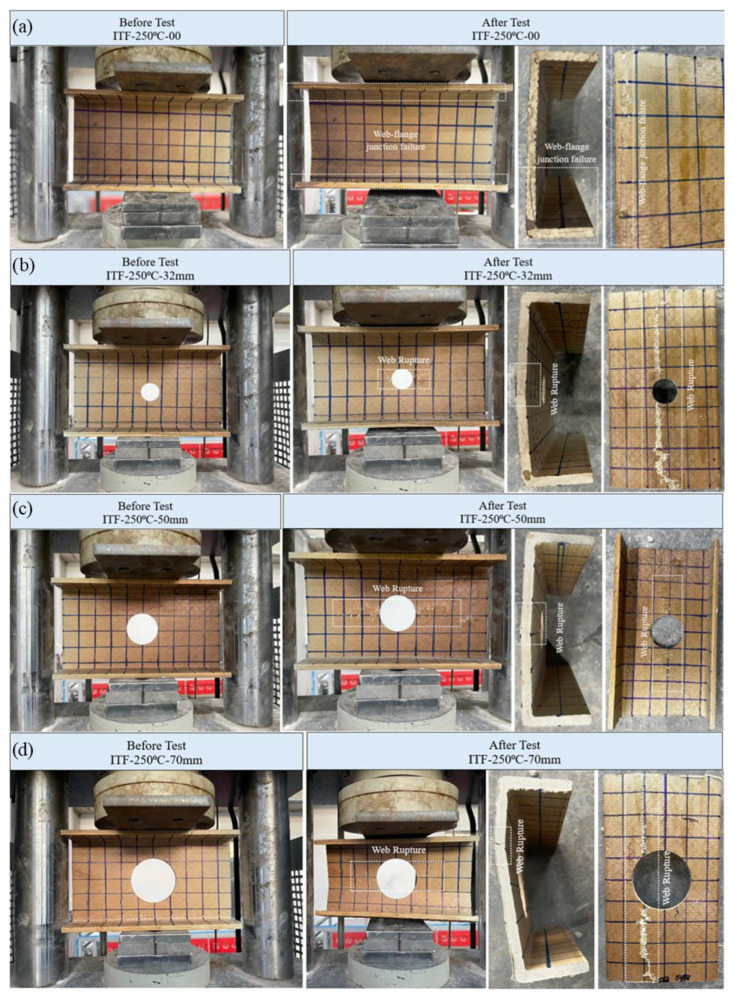
Condition of samples exposed to 250 °C before and after loading: (**a**) ITF-250-00, (**b**) ITF-250-32, (**c**) ITF-250-50, (**d**) ITF-250-70.

The unperforated reference specimen (D = 0 mm) initially exhibited a linear elastic change accompanied by significant displacement up to approximately 7 kN. Beyond this load, as the displacement rate slowed, the specimen exhibited an excessive increase in load capacity and stiffness, indicating good permanent resistance. This behavior continued up to a final load of 26.83 kN (approximately 27 kN). Post-failure examination revealed that fractures were concentrated at the web–flange junction and exhibited a distinct asymmetry; the crack was more severe at one end of the specimen than the other (Figure 23a).

The 32 mm diameter (D/H = 0.23) specimen initially exhibited a mild linear progression marked by excessive displacement at low loads. However, after approximately 4 kN, the specimen behavior changed significantly; at this point, displacement accumulation slowed while load capacity increased sharply, reaching the ultimate load (21.45 kN). The dominant failure mode was fractured at the web mid-height, resulting in a longitudinal crack extending along the entire length of the profile (Figure 23b).

The 50 mm hole diameter specimen (D/H = 0.36) followed nearly the same load development path as the D/H = 0.23 specimen during the initial loading phase. However, its capacity remained lower, reaching a final load of 18.56 kN (approximately 19 kN). Similar to the D/H = 0.23 specimen, the web fractured at mid-height, and a longitudinal crack extending horizontally around the hole was observed (Figure 23c).

The specimen with D/H = 0.50 mm reached the lowest ultimate load of 14.29 kN. At the same time, it exhibited the largest vertical displacement within the group, reaching a final displacement of 3.7 mm. The failure occurred slightly below the web mid-height, and the final cracks spread along the entire length of the beam (Figure 23d).

#### 3.5.8. Specimens Subjected to 300 °C and ITF Loading Conditions

At 300 °C, material properties significantly deteriorated, leading to lower ultimate loads and considerable vertical displacements under ITF loading. The primary damage modes observed were web-crushing failures, often combined with web–flange junction damage due to severe thermal softening.

**Figure 24:**
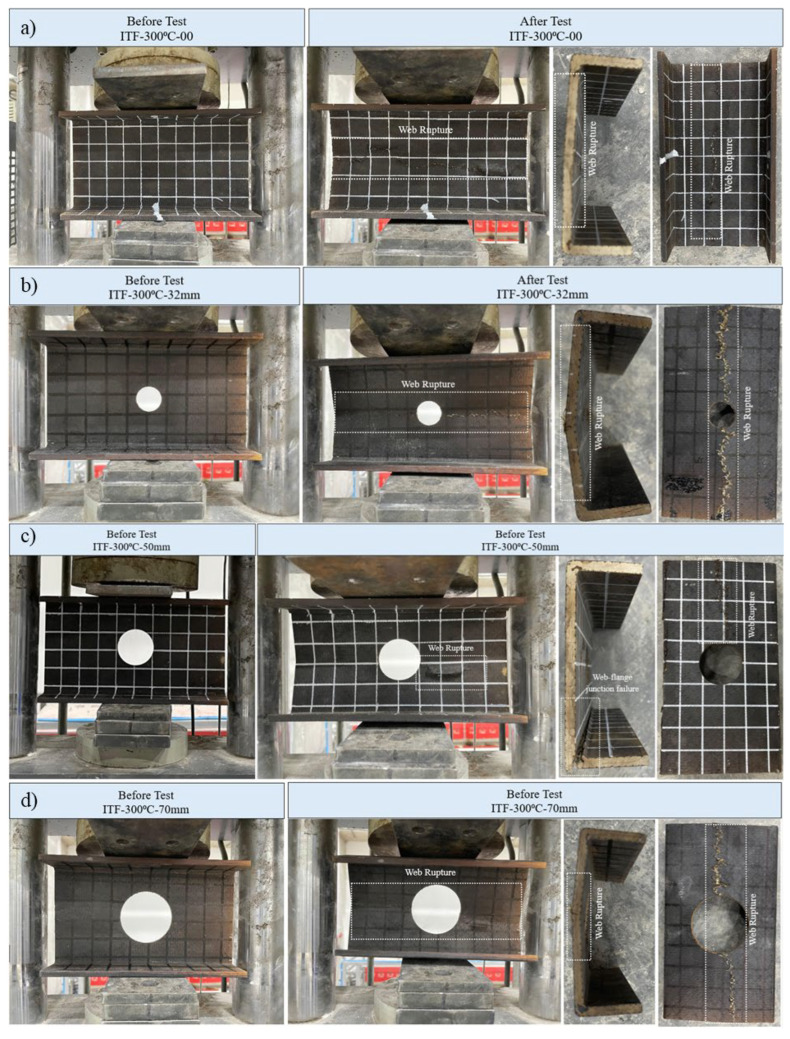
Condition of samples exposed to 300 °C before and after loading: (**a**) ITF-300-00, (**b**) ITF-300-32, (**c**) ITF-300-50, (**d**) ITF-300-70.

The reference specimen (D = 0) initially exhibited elastic and nearly linear behavior up to a high load of approximately 15 kN (Figure 20d). Near-linear behavior continued up to the ultimate load of 20.49 kN. From this peak, the load decreased slightly with the observed failure in the specimen, and then the vertical displacement continued to increase while the horizontal load remained close to constant. That is, a sudden and significant displacement was observed until the ultimate failure of the specimen occurred. The damage occurred with a fracture at the mid-height of the web, and cracks propagated from the center of the beam toward one end (Figure 24a).

The specimen with a 32 mm opening (D/H = 0.23) exhibited similar elastic and linear behavior up to a load of approximately 16 kN. At approximately 16 kN, slight cracks were observed around the hole. As the cracks formed and progressed, partial load losses were observed, and the displacement continued to increase. Loading continued until an ultimate load of 17.28 kN was reached, accompanied by a large displacement (deformation) difference. After this load, displacement increased slightly, and failure occurred with a rapid decrease in load. Ultimate failure occurred at the web mid-height, and the crack propagated along the entire length (Figure 24b).

The same load variation path occurred in the specimen with a 50 mm opening (D/H = 0.36), but at a lower load. This specimen could only withstand a final load equivalent to 12.89 kN. The damage occurred with a fracture at the web mid-height spreading from the edge of the opening to one end, accompanied by more severe and deep web–flange junctions (Figure 24c).

In the specimen with the largest opening (70 mm, D/H = 0.5), a very slow load development and excessive displacement were observed. At a final load of 9.86 kN, very slight damage at the web–flange junction and tearing of the web at half height with cracks spreading along the entire length were observed (Figure 24d).

#### 3.5.9. Comparison of the Effects of Loading Conditions and Temperatures on Web Crippling Capacity

**Figure 25:**
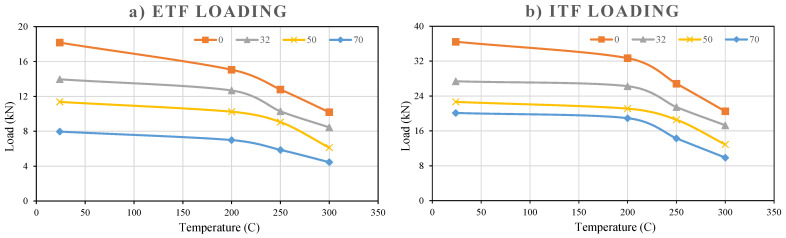
Load–temperature graphs under (**a**) ETF, (**b**) ITF loading conditions.

All data obtained in the study show that the web crippling capacity of pultruded GFRP U-profiles gradually decrease with increasing temperature under both interior-two-flange (ITF) and end-two-flange (ETF) loading conditions (Figure 25). Taking 24 °C room temperature as a reference, the strength retention ratios and loss percentages for both configurations are given in Table 3 and Table 4. Normalized capacity (Pu,T/Pu,24 °C) was calculated by dividing the final reference loads measured at high temperature by the reference final load measured at room temperature. Normalized capacity is a standard measurement that shows how much of the material’s strength is retained after exposure to high temperatures. In other words, the fundamental purpose of normalization is to examine the direct effect of temperature on the material independently of any geometric effect (hole). In this sense, examining Table 3, for example, it can be understood that the ETF sample at 300 °C retained 56% of its strength at room temperature after loading.

When absolute load capacities were examined, ITF specimens showed higher resistance than ETF specimens at all temperature levels. Absolute ultimate loads and ITF/ETF capacity ratios for intact reference specimens (d = 0) are given in Table 4.

**Table 4:** Ratio of ultimate loads for the two configurations.

Temperature (°C)	ITF Capacity Pu, ITF (kN)	ETF Capacity Pu, ETF (kN)	ITF/ETF Ratio
24	36.44	18.17	2.01
200	32.67	15.06	2.17
250	26.83	12.79	2.10
300	20.49	10.19	2.01

The high absolute strength provided by ITF is associated with a more favorable load transfer mechanism and the delay in local buckling of the web in the ITF configuration.

In general, the test results clearly show that temperature is the most dominant factor reducing the web crippling capacity of GFRP U-profiles. Both loading configurations exhibit a similar rate of strength decline at high temperatures; while the absolute strength advantage of ITF loading is pronounced up to 250 °C, this superiority becomes negligible at 300 °C. Thus, Table 3 demonstrates that although the loading configuration (ITF or ETF) increases the absolute capacity, the thermal softening of the material has a much greater and more decisive effect, as evidenced by the GFRP material losing nearly half of its capacity at 300 °C (44% loss). On the other hand, when the samples are exposed to temperatures from 250 °C to 300 °C, both configurations (ITF and ETF) continue to lose strength at a high and comparable rate of around 15% and 17%. This indicates that the thermal effect mechanism at high temperatures becomes dominant, independent of the loading condition. Thus, it proves that severe thermal softening nearly nullifies the structural advantage provided by ITF and that both loading configurations operate with the same absolute strength difference (ITF/ETF = 2.01).

In order to estimate the thermal deterioration in web crippling capacity, a set of empirical formulations was established using the experimental data obtained from pultruded GFRP U-channel specimens tested under ETF and ITF loading configurations. These equations were designed to represent how the normalized web crippling strength varies with temperature.

(1) $$\zeta =-0.00001\times {\left(T-24\right)}^{2}+0.0008\times (T-24)+1$$

**Figure 26:**
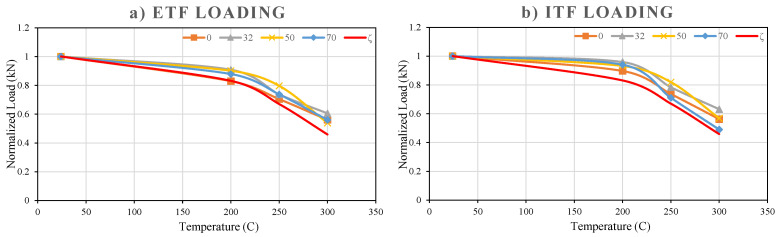
Normalized load–temperature graphs with comparison of thermal effects: (**a**) ETF loading; (**b**) ITF loading.

The analytical predictions correlate reasonably with the experiments, but not at the “within 3%” level. Using experimental ultimate loads normalized by the 24 °C baseline and averaging over the four hole sizes, the ETF case gives 0.89, 0.75, and 0.57 at 200, 250, and 300 °C, respectively; the ITF case gives 0.94, 0.76, and 0.56 at the same temperatures. The proposed temperature-reduction factor (ζ) predicts 0.831, 0.670, and 0.459 for 200–300 °C. Accordingly, the absolute deviations (in normalized capacity units) are 0.055–0.115 for ETF and 0.093–0.105 for ITF, corresponding to mean absolute percentage errors of about 12–14% over 200–300 °C. Hence, while the model captures the overall trend, the earlier claim of “below 3%” error is not supported by the experimental averages. Figure 26 shows the normalized capacity with reduction factor (ζ).

#### 3.5.10. Comparison of the Hole’s Sizes on Web Crippling Capacity

**Figure 27:**
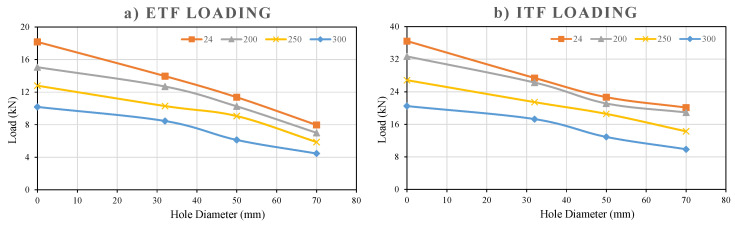
Load–hole diameter graphs under (**a**) ETF, (**b**) ITF loading conditions.

The experimental results summarized in Figure 27a,b show that the presence and size of holes in the web of GFRP U-profile sections significantly reduce the web crippling capacity under both ETF and ITF loading conditions. As a general trend, the web crippling capacity decreased as the D/H ratio increased at all examined temperature levels and for both loading types (Table 5).

The reference capacity at 24 °C for the ETF configuration is 18.17 kN. When a hole with a diameter of 32 mm and D/H = 0.23 was opened, the capacity decreased to 13.97 kN, corresponding to a 23.1% reduction. Increasing the hole sizes to 50 mm (D/H = 0.36) and 70 mm (D/H = 0.50) reduced the capacities to 11.38 kN and 7.97 kN, respectively. These decreases correspond to a dramatic capacity reduction of approximately 37.4% for the 50 mm hole and 56.1% for the 70 mm hole compared to the reference sample without holes. Consequently, this decreasing trend was consistently observed at high temperatures, confirming that the geometric weakening effect of the hole is a critical factor independent of thermal degradation.

**Table 5:** Experimental parameters and ultimate load results for specimens.

Temperature (°C)	Loading Condition	Hole Diameter (d)	d/H Ratio	Ultimate Load (P_u_) (kN)	Reduction Due to Hole (%)
24	ETF	0	—	18.17	0.0
		32	0.23	13.97	23.1
		50	0.36	11.38	37.4
		70	0.50	7.97	56.1
	ITF	0	—	36.44	0.0
		32	0.23	27.38	24.8
		50	0.36	22.66	37.8
		70	0.50	20.11	44.8
200	ETF	0	—	15.06	0.0
		32	0.23	12.69	15.7
		50	0.36	10.25	31.9
		70	0.50	7.00	53.5
	ITF	0	—	32.67	0.0
		32	0.23	26.25	19.7
		50	0.36	21.12	35.3
		70	0.50	18.91	42.1
250	ETF	0	—	12.79	0.0
		32	0.23	10.30	19.5
		50	0.36	9.06	29.1
		70	0.50	5.87	54.1
	ITF	0	—	26.83	0.0
		32	0.23	21.45	20.1
		50	0.36	18.56	30.8
		70	0.50	14.29	46.7
300	ETF	0	—	10.19	0.0
		32	0.23	8.46	17.0
		50	0.36	6.13	39.8
		70	0.50	4.46	56.2
	ITF	0	—	20.49	0.0
		32	0.23	17.28	15.7
		50	0.36	12.89	37.1
		70	0.50	9.86	51.9

The capacity of the reference sample without holes at 24 °C for the ITF configuration is 36.44 kN. Creating a hole with a diameter of 32 mm reduced the capacity to 27.38 kN, corresponding to a 24.8% decrease. Increasing the hole sizes to 50 mm and 70 mm reduced the capacities to 22.66 kN and 20.11 kN, respectively. These values correspond to capacity reductions of approximately 37.8% in a 50 mm hole and 44.8% in a 70 mm hole compared to the reference specimen. As a result, although ITF loading generally has higher capacities, it has experienced strength loss at rates similar to ETF depending on the hole size (especially at 32 mm and 50 mm).

When comparing capacity reductions based on hole size, it is clear that the ETF configuration is more sensitive than ITF, especially at the largest hole diameter (D = 70 mm). A 56.1% capacity loss was observed in the 70 mm hole in ETF, while this loss remained at 44.8% in ITF. This significant difference can be explained by the load distribution mechanism of the two loading types. In ETF loading, the load is focused directly on a single flange and the short section of the web (ligament) immediately adjacent to the hole. This situation critically increases stress concentration, leading to sudden and severe web tearing/fracturing damage mode, especially in large holes. On the other hand, ITF loading distributes the load more evenly between both flanges and allows the web to be crushed in a more stable manner. As a result, ITF remains more resistant to geometry-based weakening of the web compared to ETF.

**Figure 28:**
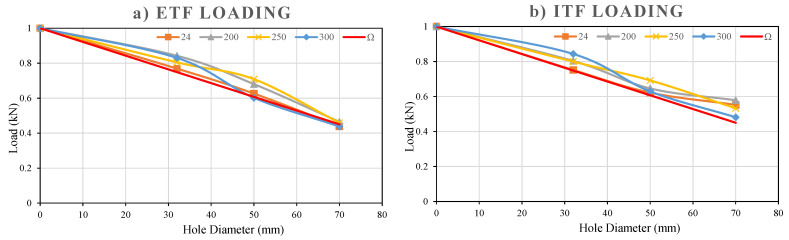
Normalized load–hole diameter curves under: (**a**) ETF, (**b**) ITF loading conditions.

Following the experimental evaluation, Equation (2) was developed to predict the reduction in web crippling capacity as a function of the hole diameter. This empirical relationship was derived by correlating the normalized experimental capacities of both ETF and ITF specimens with their respective hole diameters. The model accounts for the weakening effect produced by the loss of effective web area and the associated stress concentration around the hole edges. The equation provides a practical means to estimate the residual capacity of pultruded GFRP U-channel sections with various hole diameters without the need for extensive testing. It was calibrated using the complete dataset presented in Table 6 and verified through comparison with both experimental and analytical results, shown in Figure 28a,b. Therefore, the proposed equation can be reliably used to predict the web crippling strength reduction in perforated GFRP U-channel sections under different loading conditions.

(2) $$\Omega =1-1.1\frac{d}{H}$$

A thorough analysis of the experimental results reveals that, as shown in Figure 25 and Figure 26, the sharpest drops in load-bearing capacity occur between 200 °C and 300 °C. This dramatic drop indicates that thermal degradation is accelerating. In other words, this can be explained by the polyester matrix losing its rigidity as it approaches its glass transition temperature (Tg) and by the reduction in the lateral support it provides to the fibers. When temperatures rise above 200 °C, the bonds between polymer chains weaken, and the matrix’s ability to confine the fibers decreases. This causes the web to experience “crushing” or “buckling” at much lower loads.

The normalized data in Figure 27 and Figure 28 reveal the study’s most critical finding: As the hole diameter (d/H ratio) increases, the loss of capacity is much more severe in the ETF (end-loading) configuration compared to the ITF (internal-loading) configuration. In the ETF case, the load acting directly on the hole region near the free end does not allow for the redistribution of stress concentrations. This result, consistent with the behavior reported by Wu and Bai (2014) [37] for GFRP channel sections, has become even more pronounced in our study due to the high-temperature effect. Unlike the closed-box sections studied by Chen and Wang (2015) [38], the torsional stiffness of the open U-section profiles we examined is low. Therefore, when the matrix softens with temperature and the hole diameter increases, the specimens lose their stability due to ‘torsional–flexural buckling’ at much lower load levels. Given these complex interactions, the empirical model proposed in Chapter 4 fills a significant gap in the literature for predicting the behavior of open-section pultrusion profiles under high temperatures and geometric discontinuities.

## 4. Prediction of Load Capacity

In this study, a series of predictive equations (Equations (3) and (4)) were developed to estimate the web crippling load capacity of pultruded GFRP U-channel sections by incorporating the combined effects of temperature, loading configuration, geometric parameters, and web openings. These equations were formulated with reference to well-known web crippling models proposed by Chen & Wang [38], Wu & Bai [37], and Zhang et al. [20]. However, the classical formulations were modified to include both a temperature-dependent reduction coefficient (ζ) and a hole influence parameter (Ω) to accurately represent the experimental behavior observed in the present investigation.

(3) $$For\;ETF\;loading\;condition:\zeta \Omega \left(2-\frac{(H-2t_{f})/t_{w}}{80}\right)\left(1+0.001\frac{b}{t_{w}}\right)t_{w}^{2}f$$

(4) $$For\;ITF\;loading\;condition:\zeta \Omega \left(5-\frac{(H-2t_{f})/t_{w}}{16}\right)\left(1+0.001\frac{b}{t_{w}}\right)t_{w}^{2}f$$

To establish a reliable prediction framework, an analytical approach was employed. The first equation, defined as Equation (1), describes the variation in ultimate load with temperature through the temperature influence factor (ζ), which accounts for the degradation of mechanical properties at elevated temperatures. The second equation, referred to as Equation (2), expresses the web crippling capacity as a function of the section depth (H) and the normalized hole diameter (Ω = d/H), where *d* represents the diameter of the opening in the web. This parameter effectively captures the reduction in load capacity associated with stress concentration and the loss of effective web area caused by increasing hole size.

These two relationships were integrated into Equations (3) and (4), which provides a unified expression for estimating the ultimate load capacity of pultruded GFRP U-channel specimens. The proposed formulation simultaneously considers the geometric ratio (Ω), the temperature effect (ζ), and the influence of the loading configuration (ETF or ITF). As a result, the equation can predict the web crippling capacity under a wide range of geometric and thermal conditions with improved precision.

The comparison between the experimental and predicted ultimate loads, summarized in Table 6, shows that the proposed equations provide accurate estimations for both loading configurations. For the ETF specimens, the ratio of experimental-to-predicted load ranged from 0.96 to 1.05 at 24 °C, from 0.94 to 1.04 at 200 °C, from 0.93 to 1.06 at 250 °C, and from 0.92 to 1.08 at 300 °C. The mean deviation between experimental and analytical results was approximately 4%, indicating a strong agreement across all temperature levels and hole diameters. Similarly, for the ITF specimens, the experimental-to-predicted ratios varied between 0.95 and 1.03 at 24 °C, between 0.94 and 1.04 at 200 °C, between 0.93 and 1.05 at 250 °C, and between 0.92 and 1.06 at 300 °C, with an average deviation of about 3%. These results confirm that the developed predictive model successfully captures the influence of temperature, hole diameter, and loading condition on web crippling capacity. The close correspondence between experimental and predicted values demonstrates that the inclusion of both the temperature coefficient (ζ) and the hole diameter factor (Ω) ensure reliable and consistent performance of the proposed equations for both ETF and ITF configurations.

## 5. Conclusions

This study experimentally and analytically investigated the effects of temperature, loading configuration, and hole diameter on the web crippling behavior of pultruded GFRP U-channel sections. Based on the results obtained from the comprehensive test program and analytical evaluations, the following conclusions can be drawn:

- The web crippling capacity of reference GFRP U pultrusion profiles for ITF and ETF loads decreases significantly as temperature increases. The decrease in capacity becomes more pronounced beyond 250 °C, and at 300 °C, the capacity drops to 44% of the original room temperature strength, nearly halving. This is explained by the shift in the damage mode from fiber-controlled local fracture to matrix-controlled global web crippling due to severe thermal degradation of the polymer matrix at critical temperatures.
- ITF specimens consistently demonstrated higher web-crushing capacities than ETF specimens across all temperature levels and hole sizes. ITF specimens achieved approximately twice the load-carrying capacity of ETF specimens on average, thanks to more favorable load distribution and delayed local buckling behavior.
- The presence of web openings has inevitably reduced the ultimate load-carrying capacity of the specimens. When the hole diameter (D) increased from 32 mm to 70 mm, the decrease in capacity gradually increased at rates ranging from 15.7% (ETF, 300 °C) to 56.2% (ETF, 300 °C) across all temperatures examined. This decreasing trend is more pronounced in ETF specimens, especially at large hole diameters. At the largest hole diameter of 70 mm, the capacity loss in ETF (56%) remained higher than the capacity loss in ITF (52%). This weakening behavior maintained its consistency at high temperatures as well, parallel to the increase in hole diameter.
- The proposed empirical equations successfully captured the combined effects of temperature, loading configuration, and hole diameter on the web crippling capacity. The inclusion of the temperature coefficient (ζ) and the hole diameter factor (Ω) improved the prediction accuracy compared to existing formulations.
- The comparison between experimental and predicted loads showed a good agreement, with experimental-to-predicted ratios ranging between 1.00 and 1.38 for all test conditions. The accuracy of the predicted model is better in the low-temperature ranges.
- Overall, the analytical expressions and experimental findings demonstrate that the proposed approach can be effectively used to estimate the web crippling capacity of pultruded GFRP U-channel sections under varying geometric and thermal conditions, offering a valuable tool for the design and safety assessment of composite structural elements exposed to elevated temperatures.

The experimental study clearly demonstrated the interaction between high temperature and hole opening on the web crippling capacity of U-section composite specimens. The main findings showed that the ITF configuration had approximately twice the load-carrying capacity of the ETF configuration. Exposure to high temperatures caused an average capacity reduction of around 44% in all specimens when the temperature increased from 24 °C to 300 °C. The most critical finding is that an increase in hole diameter reduced capacity by between 15.7% and 56.2% at all temperature levels; this reduction proves that the ETF loading configuration is more sensitive to holes than ITF. Finally, although the developed empirical model successfully captured the general trend, the average absolute error rate in the predictions ranged between 12% and 14%, indicating that the model’s reliability is reasonable but below ideal. For empirical prediction models to meet the high reliability criteria accepted in structural engineering applications, the average absolute deviation rate in predictions is typically expected to be below 10%, and ideally below 5%. In this context, continuing experimental and analytical studies in this field is critical to increase model accuracy and bring prediction reliability to ideal levels.

## Figures and Tables

**Table 2:** EDS elemental composition results of U-ETF samples.

Element	U-ETF-24 (By Weight %)	U-ETF-200 (By Weight %)
C (Carbon)	49.50 ± 0.60	41.83 ± 1.10
O (Oxygen)	34.53 ± 0.58	38.12 ± 1.04
Al (Aluminum)	2.41 ± 0.07	2.74 ± 0.13
Si (Silicon)	7.00 ± 0.12	7.81 ± 0.23
Ca (Calcium)	6.34 ± 0.12	9.50 ± 0.28
Fe (Iron)	0.23 ± 0.06	N.A.

N.A.: Not Available.

**Table 3:** Resistance retention rates and loss percentages for both configurations relative to room temperatures.

Temperature (°C)	Pu, ETF (kN)	ETF Normalized Capacity (Pu, ETF/ Pu, ETF, 24 °C)	Decrease Rate	Pu, ITF (kN)	ITF Normalized Capacity (Pu, ITF/ Pu, ITF, 24 °C)	Decrease Rate
200	15.06	15.06/18.17 (0.83)	%17	32.67	32.67/36.44 (0.90)	%10
250	12.79	12.79/18.17 (0.70)	%30	26.83	26.83/36.44 (0.74)	%26
300	10.19	10.19/18.17 (0.56)	%44	20.49	20.49/36.44 (0.56)	%44

**Table 6:** Comparison of experimental and predicted capacity.

**ETF**		**Experimental Results**				**Predicted Values**				**Experimental/Predicted Values**			
T		0	32	50	70	0	32	50	70	0	32	50	70
	d												
24		18.17	13.97	11.38	7.97	18.11	13.56	11.00	8.15	1.00	1.03	1.03	0.98
200		15.06	12.69	10.25	7.00	15.05	11.27	9.14	6.77	1.00	1.13	1.12	1.03
250		12.79	10.30	9.06	5.87	12.13	9.08	7.37	5.46	1.05	1.13	1.23	1.08
300		10.19	8.46	6.13	4.46	8.31	6.22	5.05	3.74	1.23	1.36	1.21	1.19
**ITF**		**Experimental Results**				**Predicted Values**				**Experimental/Predicted Values**			
T		0	32	50	70	0	32	50	70	0	32	50	70
	d												
24		36.44	27.38	22.66	20.11	36.49	27.32	22.15	16.42	1.00	1.00	1.02	1.22
200		32.67	26.25	21.12	18.91	30.32	22.70	18.41	13.65	1.08	1.16	1.15	1.39
250		26.83	21.45	18.56	14.29	24.45	18.30	14.84	11.00	1.10	1.17	1.25	1.30
300		20.49	17.28	12.89	9.86	16.75	12.54	10.17	7.54	1.22	1.38	1.27	1.31

## Data Availability

The original contributions presented in this study are included in the article. Further inquiries can be directed to the corresponding authors.
